# *FOXC2* and *CLIP4 : a potential biomarker for* synchronous metastasis of ≤7-cm clear cell renal cell carcinomas

**DOI:** 10.18632/oncotarget.9842

**Published:** 2016-06-06

**Authors:** Jinwoo Ahn, Kyung Seok Han, Jun Hyeok Heo, Duhee Bang, You Hyun Kang, Hyun A. Jin, Sung Joon Hong, Ji Hyun Lee, Won Sik Ham

**Affiliations:** ^1^ Department of Chemistry, Yonsei University, Seoul, Korea; ^2^ Department of Urology, Seoul National University Bundang Hospital, Seongnam, Gyeonggi-do, Korea; ^3^ Department of Urology and Urological Science Institute, Yonsei University College of Medicine, Seoul, Korea; ^4^ Brain Korea 21 PLUS Project for Medical Science, Yonsei University College of Medicine, Seoul, Korea; ^5^ Department of Clinical Pharmacology and Therapeutics, College of Medicine, Kyung Hee University, Seoul, Korea

**Keywords:** clear cell renal cell carcinoma (ccRCC), whole exome sequencing, metastasis, FOXC2, CLIP4

## Abstract

Renal cell carcinomas (RCC) smaller than 7-cm are heterogeneous and exhibit metastatic potential in approximately 15% of cases. Although large-scale characterization of mutations in clear cell RCC (ccRCC), the most common RCC subtype, has been established, the genetic alterations related to ≤7-cm ccRCCs undergoing synchronous metastasis are poorly understood. To discover biomarkers that can be used to estimate the risk of synchronous metastasis in these ccRCC patients, we performed whole exome sequencing on the formalin-fixed paraffin-embedded (FFPE) samples of 10 ccRCC patients with ≤7-cm tumors and synchronous metastasis and expanded our study using The Cancer Genome Atlas (TCGA) ccRCC dataset (n = 201). Recurrent mutations were selected according to functional prediction and statistical significance. Mutations in three candidate genes, *RELN* (1 out of 10), *FOXC2* (1 out of 10), and *CLIP4* (2 out of 10) were found in expanded analysis using a TCGA cohort. Furthermore, siRNA-mediated target gene knockdown (*FOXC2* and *CLIP4*) and overexpression (*RELN*) assays showed that FOXC2 and CLIP4 significantly increased cell migration and viability in ccRCCs. Our study demonstrated that FOXC2 and CLIP4 activity correlates to the presence of ≤7-cm ccRCCs with synchronous metastasis and may be potential molecular predictors of synchronous metastasis of ≤7-cm ccRCCs.

## INTRODUCTION

Diagnoses of early-stage renal cell carcinomas (RCC) smaller than 7-cm have increased in incidence, likely due to more widespread use of cross-sectional imaging [1]. These lesions were previously presumed to be aggressively malignant and managed with radical or partial nephrectomy. However, we now recognize they frequently exhibit favorable pathology and prognosis [2], and therefore, non-surgical approaches, such as cryoablation, radiofrequency ablation, and active surveillance are valuable alternative treatments for a subset of patients, e.g. elderly patients, those with single kidney, or those deemed unfit to undergo a major surgery due to comorbidities [3–4].

However, ≤7-cm RCCs demonstrate great heterogeneity and exhibit metastatic potential in approximately 15% of cases [5–7]. Thus, early determination of prognosis in these patients is critical for selecting the proper treatment, tailoring the patients' clinical surveillance, and effectively allocating patients into clinical trials. Although the TNM staging system is used to predict patient outcomes, each stage is comprised of heterogenous populations [6]. Moreover, no preoperative diagnostic test is available to predict the biological behavior of these RCCs. Tumor size is currently the best preoperative prognostic parameter, although it cannot accurately indicate renal tumor aggressiveness [8]. Some renal tumors ≤7-cm are preoperatively staged highly based on radiographic suspicion of the tumors extending into the renal vein, its branches, or perinephric and/or renal sinus fat, but with limited accuracy [9]. The recent use of percutanenous renal mass biopsy has increased considerably to address the growing incidence of ≤7-cm RCCs and the need for early diagnoses to facilitate effective treatment [10–11]. Although the biopsy can be safely performed with image-guided equipment, the procedure still lacks accuracy of histologic subtyping (88 to 94%) and grading (64 to 70%) [12–13]. Therefore, identifying novel molecular biomarkers from renal mass biopsy sample, which can indicate the metastatic potential of these RCCs are needed to accurately stratify patients for treatments less invasive than surgical excision and to minimize risk of under-treatment.

Molecular biomarkers have been shown to aid the diagnoses and disease monitoring efforts for several cancers, but such markers are not currently available for RCC [14]. Recently, The Cancer Genome Atlas (TCGA) has published research on RCC, and additional RCC data from many other studies have been uploaded to the TCGA data portal. Several studies of RCC samples identified genes, including *VHL*, *PBRM1*, *PIK3CA*, and *MLL2*, that are recurrently associated with RCCs [15]. Some genes associated with metastasis in RCC are known, but because these analyses usually focus on identifying recurrently mutated genes regardless of tumor size, it is unclear whether these genes are associated with the metastatic potential of ≤7-cm RCCs. Thus, common genetic alterations found in previous studies cannot identify the genetic mutations that drive synchronous metastasis in these tumors.

Here, we performed whole exome sequencing of samples from patients with clinical stage T1 (tumor size ≤7-cm) clear cell RCC (ccRCC), the most common RCC subtype, that exhibit synchronous metastasis. We also performed a gene set comparison with the RCC data from TCGA to identify candidate genes associated with synchronous metastasis in these ccRCCs. These analyses revealed that *Reelin (RELN), Cytoskeleton-associated protein – glycine rich (CAP-Gly) domain-containing linker protein family member 4 (CLIP4)*, and *Forkhead box protein C2* (*FOXC2)* are frequently mutated in these patients and represent metastasis-associated genes.

Quantitative reverse transcriptase PCR (qRT-PCR) of a panel of RCC cell lines revealed that *FOXC2* was relatively highly expressed only in the metastatic Caki-1 and UMRC-3 cell lines compared to other cell lines. *CLIP4* exhibited high fold-changes in expression levels in all kidney cancer cell lines compared to normal kidney cell lines. However, *RELN* was not expressed in any RCC cell lines. Focusing on *FOXC2* and *CLIP4,* we performed siRNA-mediated target gene knockdown, which revealed that *FOXC2* and *CLIP4* knockdown are associated with decreased cell invasion and migration in ccRCC cell lines. In contrast, when overexpressed, *RELN* only increased cell viability, not cell invasion or migration. Our research provides an integrated approach for identifying novel genes that exhibit functional roles in metastasis in RCC cell lines and are associated with synchronous metastasis in ≤7-cm ccRCCs, therefore they could be used as new potential biomarker to predict the metastatic potential in these tumors.

## RESULTS

### Exome sequencing of ≤7-cm ccRCCs exhibiting synchronous metastasis

Ten formalin-fixed paraffin-embedded (FFPE) samples of ≤7-cm ccRCCs exhibiting synchronous metastasis were eligible for whole exome sequencing analysis. We obtained the clinical and pathological details of each study patient (Table 1 and Supplementary Table S1) and collected tumor and normal samples with average depths of 81× and 58× with 90.5% and 85.3% coverage of the target region, respectively, using, on average, greater than 20 non-duplicated reads per sample (Supplementary Table S2). Although all 10 tumors were staged T1 due to their size, 30% of the tumors were pathologically staged T3 due to renal sinus fat invasion.

**Table 1:** Clinical status of the ccRCC exome sequenced patients (tumor size ≤7-cm) and The Cancer Genome Atlas

	ccRCC Exome patients	TCGA ccRCC patients
***Characteristics***		
No. of patients	10	201
Age, median (range)	56 (45 ~ 72)	59 (35 ~ 90)
Gender, n (%)		
Male	7 (70.0)	130 (64.7)
Female	3 (30.0)	71 (35.3)
***Pathological stage, n (%)***		
T1	7 (70.0)	149 (74)
T3	3 (30.0)	51 (25)
T4	0 (0.0)	1 (1)
***Ethnic group, n (%)***		
Caucasian	0 (0.0)	187 (93.0)
Asian	10 (100.0)	4 (2.0)
African	0 (0.0)	7 (3.5)
n/a	0 (0.0)	3 (1.5)
***Exome Mutations***		
Median nonsynonymous somatic mutations per Mb (range)	0.78 (0.13 ~ 1.27)	2.1 (0.57~6.37)
Mean nonsynonymoussomatic mutations per Mb (SD)	0.73 (0.35)	2.3 (0.96)

No., Number; n/a, Not Available;

SD, Standard Deviation.

**Figure 1:**
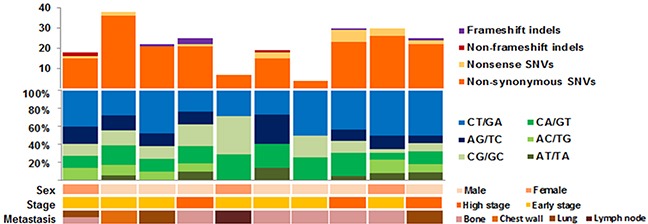
Mutation profile for our exome sequenced synchronous metastasis of ≤7-cm clear cell renal cell carcinoma cohort

After somatic exonic mutation calling was performed, synonymous mutations were rejected from further analysis. To reconcile the overall true-positive rate, 10% of the total somatic variant candidates (24 variants) were validated with Sanger sequencing, which showed a 95.8% (23 out of 24) concordance rate (Supplementary Table S3). Overall, 209 somatic single nucleotide variants (SNVs) and nine somatic indels were identified from 10 samples (Supplementary Table S4), corresponding to 0.73 mutations per megabase (Supplementary Table S5). By grouping each somatic SNV according to its nucleotide change, the average ratio of transitions to transversions was 1.16, where C:G > T:A transition mutations were the most common mutations (48.7%) identified in our dataset, followed by C:G > A:T transversions (23.3%) (Figure 1). These findings are consistent with previous ccRCC studies [16].

**Figure 2:**
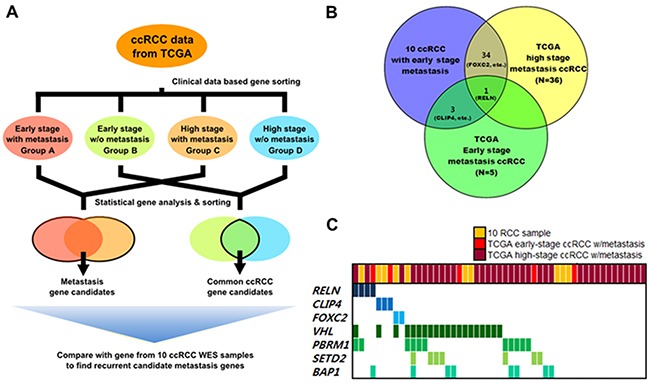
Analysis flow and results of metastasis-associated gene selection Clinically early stage indicates patients with renal tumors ≤7-cm and clinically high stage indicates patients with renal tumors stage ≥T3. **A.** Overview of expanded analysis using the TCGA cohort to identify metastasis-associated genes in ccRCC. **B.** Frequently mutated candidate metastasis-associated genes identified from comparison analysis. **C.** Gene profiles across ccRCC samples with metastasis used in analyses.

Validated candidate mutations were selected based on four functional prediction programs (Polyphen, SIFT (Sorting Intolerant From Tolerant), Mutation Taster, and LRP (Likelihood Ratio Test)). Mutations predicted to be critical (defined as likely damaging or deleterious mutations) in at least one prediction program were considered valid mutations and used for further analyses. Genes previously known to be frequently mutated in ccRCC [15], including *VHL*, *PBRM1*, *MLL2,* and *ZNF536* were also found in our dataset, wherein *VHL* was the most frequently mutated gene (50%, 5 out of 10) (Figure 2). Other genes not frequently mutated in cases of ccRCC, were further analyzed to identify metastasis-associated genes.

### Expanded data analysis using TCGA dataset

Due to the small number of FFPE samples, we collected additional data on ccRCC using the dataset from TCGA and performed expanded data analysis to select candidate genes that contribute to synchronous metastasis of small ccRCCs. By focusing on exonic somatic mutations, samples with at least one non-synonymous mutation or a splicing mutation were extracted for analysis. The median non-synonymous mutation rate of the expanded TCGA dataset was 2.1 mutations per megabase with an average standard deviation of 0.96. C:G > T:A transitions were the most frequently observed mutations (31%), and the average ratio of transitions to transversions was 1.00, similar to our exome sequencing dataset.

Overall, 201 ccRCC patients were included for expanded analysis. We collected demographic and clinical information of each patient, including age, gender, tumor stage, and ethnicity (Table 1). We separated the ccRCC samples from the expanded TCGA data pool into four categories according to pathological stage and presence of metastasis. Group A was composed of patients with early-stage ≤7-cm ccRCCs with synchronous metastasis (n = 5) similar to our FFPE samples. Group B included patients with early-stage ≤7-cm ccRCCs without metastasis (n = 144). Groups C and D consisted of patients with late-stage (≥T3) tumors with metastasis (n = 36) or without metastasis (n = 16), respectively.

Genes that were commonly mutated in groups without metastasis (groups B and D) were considered to be less associated with metastasis and rejected for further gene selection (Figure 2A). For the remaining genes with mutated loci in our dataset (Groups A and C), we analyzed the amino acid changes and functional predictions (Supplementary Table S6). Among these candidate genes, many were previously known to induce tumor metastasis in other types of cancers [17–19]. However, a total of three genes were found to be significantly associated with metastasis (Figure 2B).

One of the selected genes, *RELN*, is a well-known regulator of corticogenesis, and influences autism, but recently *RELN* was found to be associated with metastasis in several types of cancer, such as pancreatic cancer [17] and esophageal cancer [18]. In our dataset, a mutation in *RELN* was found in one sample (1/10, 10%) in group A (1/5, 20%) and two samples in group D (2/36, 20%) (Table 2). Another candidate gene, *FOXC2* (1/10, 10%), is known to be associated with development and many other cellular processes. Moreover, recent *in vitro* assays using shRNA revealed roles for *FOXC2* in breast cancer metastasis [19]. Prior to this study, link between *CLIP4* and tumor metastasis had not yet been clearly identified. However, CLIP4 expression was found to inhibit c-Cbl to suppress EGFR signaling [20] which has association with EMT signaling [21]. Also, two samples from our dataset harbored *CLIP4* mutations and one sample from group A contained a mutation in *CLIP4* that was enriched in ccRCC samples with synchronous metastasis. In our analyses, these three candidate genes were significantly mutated (*p* ≤0.05, calculated with MutSigCV) in metastatic ccRCC samples. Moreover, we performed Sanger sequencing of the *RELN*, *FOXC2*, and *CLIP4* mutations and validated the mutations in these three genes as true-positive mutations.

**Table 2:** Mutated loci and mutation frequencies for the candidate metastasis-associated genes from our cohort and the TCGA cohort

Gene symbol	Gene name	Mutation frequency			Total (n)	AA substitution	No. of damage algorithms
		Exome sequencing set (n = 10)	TCGA group A (n = 5)	TCGA group C (n = 36)			
*CLIP4*	CAP-GLY domain containing linker protein family member 4	2/10	1/5	-	3/51	G286E	4/4
						S433C	2/4
						G483E	1/4
*FOXC2*	Fork head box C2 (Mesenchyme forkhead 1)	1/10	-	1/36	2/51	T230R	1/2
						G43D	3/3
*RELN*	Reelin	1/10	1/5	2/36	4/51	F1998V	4/4
						A535P	3/4
						P280T	4/4
						R2428W	4/4

AA, amino acid; No., number.

### Candidate gene expression pattern analysis in kidney cells

To validate the functional roles of these genes in metastasis, six kidney cell lines were selected for *in vitro* studies. Hk-2 cells were selected as the normal kidney cell line, and five cell lines (786-0, A498, Caki-1, UMRC-3, and UMRC-6) were selected to represent kidney cancer. qRT-PCR was performed on all six cell lines for the three candidate genes (*RELN*, *FOXC2*, and *CLIP4*) to identify those with significant fold-changes in gene expression in the kidney cancer cell lines compared to the normal kidney cell line (Supplementary Figure S1). According to our analyses, *FOXC2* exhibited the most significant change in expression among the three candidate genes. *FOXC2* expression was significantly increased in Caki-1 and UMRC-3 cells, which are metastatic cell lines, compared to other cells. Therefore, overexpression of *FOXC2* was considered to be associated with kidney cancer metastasis and was chosen for further gene knockdown studies. *CLIP4* demonstrated high fold-changes in all kidney cancer cell lines compared to the normal kidney cell line. In contrast, we failed to detect expression of *RELN* in the kidney cell lines and could not extrapolate an expression pattern specific to metastatic kidney cells. Thus, despite *RELN* being the most frequently mutated gene in our analysis, and epigenetic silencing of *RELN* being previously shown in several cancers, we decided to pursue *RELN* as a candidate gene.

### Knock-down of *FOXC2* and *CLIP4* inhibits cell migration in RCC cells

**Figure 3:**
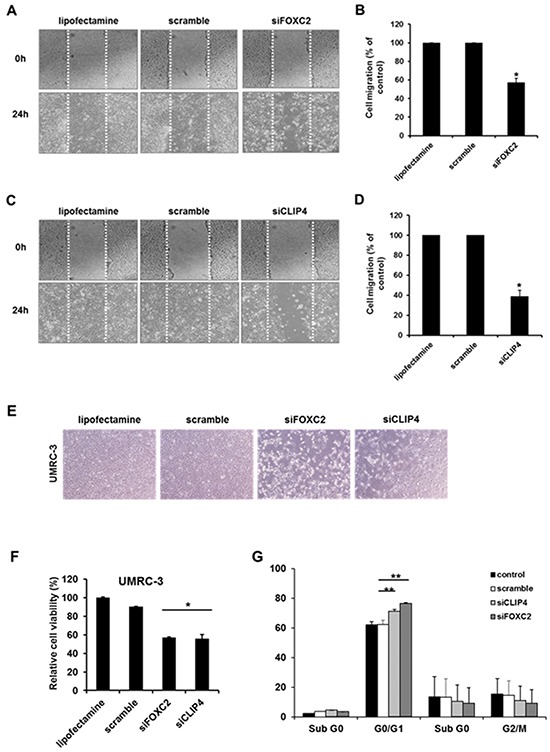
*In vitro* cell migration, proliferation and viability assay of candidate genes (*CLIP4* and *FOXC2*) with siRNA gene knock-down **A.** Representative images of cell migration in the wound-healing assay targeting *FOXC2*. **B.** Relative cell migration ratios are presented as the mean ± sd (n = 3; *, *p* <0.05). **C.** Representative images of cell migration in the wound-healing assay targeting *FOXC2*. **D.** Relative cell migration ratios are presented as the mean ± sd (n = 3; *, *p* <0.05). **E.** Representative images of the changes in cell proliferation after siRNA-mediated target gene knockdown. **F.** Relative changes in cell viability after siRNA-mediated target gene knockdown (n = 3; *, *p* <0.05). **G.** Quantification of the cell populations in various stages of the cell cycle using fluorescence activated cell sorting (FACS) flow cytometry with propidium iodide (PI) staining (n = 3; **, p <0.01).

To further confirm the roles of *FOXC2* and *CLIP4* in RCC cells, we performed *in vitro* gene knockdown assays. We transfected siRNA targeting *FOXC2* or *CLIP4* into the metastatic RCC cell line, UMRC-3, for functional validation. GAPDH siRNA and scrambled siRNA were used as controls, and we performed wound-healing assays as a proxy for cell migration. *FOXC2* and *CLIP4* knockdown in UMRC-3 cells led to significantly reduced cell migration compared to control cells (Figure 3A–3D). Additionally, further experiments with MTT (3-(4,5-dimethylthiazol-2-yl)-2,5-diphenyltetrazolium bromide) showed that knockdown of *FOXC2* and *CLIP4* reduced cell viability by approximately 40% in UMRC-3 cells (Figure 3E, 3F) and induced G0/G1 cell cycle arrest (Figure 3G).

We also examined wound healing and cell viability when *RELN* was stably overexpressed (Supplementary Figure S2). Unlike *FOXC2* and *CLIP4*, expression of *RELN* had no effect on cell migration. In contrast, the cell viability assay demonstrated that *RELN* overexpression induces cell proliferation in RCC cells. Collectively, these data suggest that overexpression of *FOXC2* and *CLIP4* likely increases cell migration, which correlates to the synchronous metastasis of early-stage RCCs, whereas *RELN* may aid cancer cell survival.

## DISCUSSION

Identifying tumor biomarkers, such as mutated genes that promote cancer cell invasion and colonization of distant organs, can enable early and accurate determination of the patient's prognosis and the tumor's pathologic characteristics [22–23]. To date, no useful preoperative prognostic clinical factor for predicting the synchronous metastatic potential of ≤7-cm ccRCCs is available, and genes that initiate metastasis in these tumors are poorly understood. Discovering candidate genes that influence metastasis can allow for treatments less invasive than surgical excision, but without risk of under-treatment [3–4], and provide tailored surveillance and effective patient allocation into adjuvant clinical trials [24–26].

Our somatic mutation profile of ≤7-cm ccRCCs with expanded analysis using the ccRCC exome dataset in the TCGA [15] enabled us to identify candidate genes associated with synchronous metastasis in these tumors. We analyzed the mutated genes in our ccRCC dataset and TCGA early- and late-stage metastatic tumors to determine when late-stage tumor mutations affected the tumors' metastatic potential, although diagnosis and surgery were performed during the late stage. Our results highlight that *FOXC2* and *CLIP4* are critical genes impacting the metastatic potential of ≤7-cm ccRCCs.

FOXC2 is an evolutionarily highly conserved helix transcription factor with a DNA binding domain [27] that is necessary for embryonic development and many other key biological processes [28–29]. Processes such as embryonic development and metastasis are highly reliant on the migratory ability of cells [30–31]. In our analysis, *FOXC2* was mutated in 1/10 samples in our dataset, and mutations in *FOXC2* were also found in TCGA high-stage with metastasis group (2.8%, 1/36). With pathway analysis using the DAVID functional annotation tool (http://david.abcc.ncifcrf.gov/tools.jsp), *FOXC2* was found to be correlated to cancer-associated pathways, including the TGF-b [32], Notch [33], ERK and Wnt signaling pathways [34–35] (Supplementary Figure S3A).

FOXC2 was previously termed Mesenchyme Forkhead 1 (MFH-1), and recently has been identified as an essential mediator of the epithelial-mesenchymal transition (EMT) process [36–38] that is mainly activated during tumor metastasis and progression [39–40]. EMT arises from various developmental processes and is triggered by several signals including the Wnt and TGF-β signaling pathways [35, 41]. Induction of EMT could weaken cell-cell interactions and cell polarity, which can influence the cytoskeleton and facilitate increased cell motility and invasiveness [42]. Several studies confirmed that *FOXC2* mRNA and protein expression are induced by EMT-inducing factors (Snail, Twist, Goosecoid, Slug, SIP1, and E12/E47), which was discovered in various types of cancers [19, 43].

Some of the downstream targets of FOXC2 have been characterized as well. FOXC2 specifically suppresses E-cadherin and induces the expression of matrix metalloproteinases in breast cancer to promote metastasis [19]. Additionally, *FOXC2* can inhibit p120-catenin and suppress E-cadherin [44], which supports the reported role of *FOXC2* in metastasis.

Our *in vitro* studies revealed that *FOXC2* expression increases cell migration in ccRCC patients. Additionally, *FOXC2* is known to induce cell proliferation and angiogenesis, which gives cancer cells stem cell-like properties [38]. This finding is consistent with other reported functional roles of *FOXC2* in different types of cancers [19, 43]. Altogether, these previous findings demonstrate that *FOXC2* expression is correlated with increased cell invasiveness and viability, which can promote synchronous metastasis in early-stage small ccRCCs.

CLIP4, also known as UBASH3A or TULA, is a member of the T-cell ubiquitin ligand family and suppresses T-cell signaling. CLIP4 can facilitate growth factor withdrawal-induced apoptosis in T-cells [45] and promote the accumulation of activated various target receptors, such as T-cell receptors, epidermal growth factor receptor, and platelet-derived growth factor beta-receptor [20, 46], which can induce cell invasiveness and metastasis. CLIP4 were found to activate Syk [47], one of the protein tyrosine kinase, and Syk expression were known to be linked with cell motility and increased cell migration [48–49]. Also CLIP4 found to be capable of inhibiting c-Cbl which downregulates EGF receptor and activate EGFR signaling pathway [20] which is known to be associated with EMT pathway [21] (Supplementary Figure S3B). Close family members of *CLIP4/UBASH3B* were also found to be associated with metastasis in breast cancer [50], and the same study also suggested that *CLIP4* expression could stimulate tumor metastasis in some tumor types.

Given these findings, we assumed that the overexpression of *CLIP4* was associated with tumor metastasis in RCC. We performed *in vitro* gene knockdown studies to test this hypothesis. Consistent with our assumption, knockdown of *CLIP4* remarkably inhibited cell migration and viability. This finding is the first evidence that *CLIP4* expression could induce metastasis in ccRCC. However, considering that the details of how *CLIP4* influences tumor progression are still unclear, further research on the functional role and mechanism(s) of *CLIP4* is needed.

The limitations of our study should be acknowledged and addressed. Firstly, due to the retrospective nature of our study and the small number of FFPE samples, our conclusions are preliminary and should be confirmed in a prospective clinical setting with a larger sample size that includes only patients with pathologically-staged T1 tumors. Secondly, because tumor size is an important determinant of the likelihood of synchronous metastasis also in patients with ≤7-cm ccRCCs [51], the relationship between the tumor size and the expression of *FOXC2* and *CLIP4* should be clarified. Thirdly, intratumor heterogeneity within primary tumors and associated metastatic sites has been well known in RCCs [52]. Therefore, not only primary renal lesions but also metastatic lesions should be investigated to clarify the role of *FOXC2* and *CLIP4* in synchronous metastasis of ≤7-cm ccRCCs in further study.

In summary, our data suggest that *FOXC2* and *CLIP4* are associated with synchronous metastasis in ≤7-cm ccRCCs and could be used as potential biomarker genes from renal mass biopsy sample, which can determine the metastatic potential of these tumors could enable the application of personalized treatment strategies in ccRCC patients. Moreover, regulation of *FOXC2* and *CLIP4* expression, which could suppress cell migration and inhibit tumor metastasis, would provide future helpful anti-cancer strategies to improve the survival of patients with metastatic ccRCCs.

## MATERIALS AND METHODS

### Sample selection and DNA preparation

Among the 1,447 patients who underwent radical or partial nephrectomies for solid enhancing renal tumors at our institution between 2005 and 2014, 569 patients were diagnosed with ≤7-cm ccRCCs. Tumor size was measured by CT or MRI Patients with preoperative radiographic indications of tumor extension into the renal vein, its branches, or perinephric and/or renal sinus fat and positive postoperative surgical margins were excluded. Twelve patients presented with synchronous metastasis and were subsequently chosen as study subjects. We defined synchronous metastasis as metastasis at or within three months of the primary RCC diagnosis [53]. Of the 12 patients, FFPE tumor samples from 10 patients were available and used for the analysis. Based on the seventh edition of the American Joint Committee on Cancer staging system, additional clinical information, including gender, smoking history, and age, was collected (Supplementary Table S1). All patients provided consent for their samples to be used for research purposes. The study was approved by the Institutional Review Board of Severance Hospital and conducted in accordance with the Helsinki Declaration. DNA was extracted from FFPE tumor samples with a QIAamp DNA FFPE Tissue Kit (Qiagen, Valencia, CA, USA) and a DNeasy Tissue Kit (Qiagen, Valencia, CA, USA), respectively.

### Whole exome sequencing and data analysis

To sequence the exomes of the 10 ccRCC pairs, a SureSelect Human All Exon V5 kit (Agilent Technologies, Santa Clara, CA, USA) was used, and the samples were sequenced on an Illumina Hiseq 2500 platform (Illumina, San Diego, CA, USA) according to the manufacturer's protocols. The sequencing data were aligned to a reference genome from the National Center for Biotechnology Information, build 37 (hg19), with Novoalign (version 1.02.01) using the default options. In the process, the Genome Analysis Toolkit (GATK; version 1.4-21) was used for local realigning around indels, quality score recalibration, and mate pair fixing, and Picard was used to remove duplicated reads. The variant-calling program, Mutect (version 1.0.287783), was used with default options to call somatic SNVs, and the Somatic Indel Detector (from GATK, version 1.4-21) was used to call somatic indels using the ‘tumor-normal paired sample’ mode.

Mutated loci were sorted with the following criteria: variant allele frequency ≥10%; depth of loci ≥20; and known single nucleotide polymorphism (SNP) from the 1,000 Genomes Project, with a minor allelic frequency ≥1%. The remaining mutations were annotated with ANNOVAR, and only non-synonymous mutations in coding exonic regions were included for further analysis. Somatic indels were manually validated with Integrated Genome Viewer software (IGV; version 2.3.5) to avoid false positive indels. All of the sequence-aligned bam files generated by our pipeline (Supplementary Figure S4) are accessible on the Sequence Read Archive (accession number: SRR3056856).

### ccRCC data from the cancer genome atlas (TCGA)

To validate and collect a control gene set for ccRCCs, we used ccRCC data from the TCGA data portal. Samples with available exome data (n = 516) were downloaded after examining the pathology reports of all the patients. Patients were excluded when accompanied by other malignancies were excluded, and 201 patients were included in our gene comparison set (Supplementary Table S7). Genes that were mutated in pathologically non-metastatic ccRCC patients (n = 160) with a MutSigCV (version 1 from GenePattern, http://genepattern.broadinstitute.org) *p* ≤0.05 were considered common genes found in all ccRCCs and used as the control gene set.

### Sanger validation of detected mutations

Primer pairs for targeting selected loci were designed by the Primer3 program (Supplementary Table S3). To amplify target regions, we prepared mixtures of 1 μl of each primer (10 μM; Macrogen, Seoul, Korea), 10 μl of Taq polymerase master mix (Intron, Korea), 8 μl of distilled water, and 1 μl of target gDNA. PCR conditions were as follows: 3 min at 95°C; 30 cycles of 30 sec at 95°C, 30 sec at 60°C, and 30 sec at 72°C; and 10 min at 72°C. PCR products were purified with the QIAquick Gel Extraction Kit (Qiagen, Valencia, CA, USA). Purified products were sent for Sanger sequencing (Macrogen, Seoul, Korea), and the mutation peak analysis with Sanger sequencing data were analyzed with the SeqMan program (DNASTAR, Madison, WI, USA).

### Cell culture and reagents

The human RCC cell line, Caki-1, was obtained from the American Type Culture Collection (ATCC) and maintained in McCoy's 5A medium (Hyclone, Thermo Fisher Scientific, Waltham, MA, USA) supplemented with 10% fetal bovine serum (FBS; Sigma-Aldrich, St Louis, USA). Two additional human RCC cell lines, UMRC-3 and UMRC-6 (gifts from Dr. P. Black, Vancouver Prostate Centre, University of British Columbia, Canada), were maintained in Minimal Essential Medium (MEM; Invitrogen, Carlsbad, CA, USA) supplemented with 10% FBS and 2 mmol/L L-glutamine (Sigma-Aldrich, St Louis, USA). Cell line authentication for UMRC-3 and UMRC-6 was performed using fingerprinting and the AmplFLSTR identifier amplification kit (Applied Biosystems, Foster, CA, USA) in the Korean Cell Line Bank (Seoul, Korea). The human RCC cell line, 786-O, was purchased from the ATCC and maintained in Roswell Park Memorial Institute (RPMI)-1640 medium (Hyclone, Thermo Fisher Scientific, Waltham, MA, USA) supplemented with 10% FBS. The human renal epithelial cell line, HK-2, was purchased from the ATCC and cultured in Dulbecco's Modified Eagle's Medium (DMEM)/Ham's F12 (Invitrogen, Carlsbad, CA, USA) supplemented with 10% FBS and 2 mmol/L L-glutamine. All cells were cultured at 37°C in a humidified atmosphere with 5% CO_2_. For all experiments, cell lines were maintained for no more than 2 months.

### qRT-PCR analysis of target genes from RCC cell lines

qRT-PCR was performed on the human kidney cell lines: 786-0, A498, UMRC-3, UMRC-6, Caki-1, and Hk-2. One microgram of RNA from each target cell line was extracted using the RNA RNeasy Mini Kit (Qiagen, Valencia, CA, USA) and used for reverse transcription using Accupower ® RocketscriptTM (Bioneer, Seoul, Korea). GAPDH expression in each sample was used to normalize candidate gene expression analysis using the 2^−ddCt^ method to generate a relative standard curve.

For each reaction, the well contained 1 μl of template cDNA, 10 pmol of each target gene primer, and Accupower® Greenstar™ qPCR PreMix (Bioneer, Seoul, Korea) in a final volume of 20 μl. PCR reactions were performed using an Exicycler TM 96 Real-Time Quantitative Thermal Block with the following conditions: 10 min at 95°C; 40 cycles of 10 sec at 95°C, 30 sec at 58°C, and 30 sec at 72°C; and 5 min at 65°C. Each reaction was performed in triplicate. The primers used for qRT-PCR were *RELN–* PH01298, *FOXC2* – PH01299, *CLIP4* – PH01302, and *GAPDH –* PH00003 (Bioneer).

### Small-interfering RNAs for gene knockdown

Small-interfering RNAs (siRNAs) targeting *FOXC2* and *CLIP4* were purchased from Dharmacon (Lafayette, CO, USA). siRNA targeting *FOXC2* consisted of four different sequences: 5′-CCUACGACUGCACGAAAUA-3′, 5′-CCAACG UGCGGGAGAUGUU-3′, 5′-GGAUUGAGAACUCG ACCCU-3′, and 5′-GCGCCUAAGGACCUGGUGA-3′. siRNA targeting *CLIP4* also consisted of four sequences: 5′-UGAUAGAGAUGGAUUGACA-3′, 5′-GUAGAUA UGCCGUUAGAGA-3′, 5′-CGGAGUCGCUGGACAA ACA-3′, and 5′-AGGAUAUUGGUAUGGUAUA-3′.

Caki-1 and UMRC-3 cells were transiently transfected with the 21-bp siRNA molecules targeting *FOXC2* and *CLIP4* or a non-silencing scrambled sequence siRNA (si-Scr). Caki-1 and UMRC-3 cells were plated onto 6-well plates at a density of 1.2 × 10^5^ cells/well and then transfected at 30-50% confluency for 16 h with the siRNAs and Lipofectamine 2000 (Invitrogen, Carlsbad, CA, USA) diluted in OPTIMEM (Invitrogen, Carlsbad, CA, USA) according to the manufacturer's instructions. After transfection, the media was replaced, and the cells were incubated for 48-72 h based on the purpose of the experiment.

### Cell viability assay

Cells were seeded onto 6-well plates at a density of 1.2 × 10^5^ cells/well and incubated overnight. After transfection, cells were incubated for 72 h, fixed with 1% glutaraldehyde (Sigma-Aldrich, St Louis, USA), and stained with 0.5% crystal violet solution (Sigma-Aldrich, St Louis, USA). Cells were washed with water, and the remaining crystal violet was resolved with Sorensen's solution. Absorbance was measured at 562 nm by spectrophotometry to measure cell viability. All experiments were performed in triplicate.

### Wound-healing assay

Wound-healing assays were used to assess directional cell migration. Cells were plated onto 6-well plates and allowed to form a confluent monolayer. Wounds were made in each well using a 200-μl pipette tip. After scratching, all floating cells and debris were washed out with phosphate-buffered saline (PBS) twice, and the remaining cells were incubated in culture media for an additional 36 h. Wound healing was recorded every 6 h by microscopy. Each experiment was performed in triplicate, and, in each experiment, the area across the wound mark was determined in five microscopic fields. The percentage of wound healing was calculated using the equation: (percent wound healing) = average of ([gap length: 0 h] - [gap length: 18–24 h]) / [gap length: 0 h]).

## SUPPLEMENTARY FIGURES AND TABLES










