# Supplementary material for: FOXC2 and CLIP4 : a potential biomarker for synchronous metastasis of ≤7-cm clear cell renal cell carcinomas
Source: Oncotarget. 2016 Jun 6;7(32):51423–34. doi: 10.18632/oncotarget.9842 (PMC5239485; doi:10.18632/oncotarget.9842)
Supplement: Supplementary file 1 [file oncotarget-07-51423-s001.pdf]

FOXC2 and *CLIP4* : *a potential biomarker for* synchronous metastasis of ≤7-cm clear cell renal cell carcinomas

SUPPLEMENTARY FIGURES AND TABLES

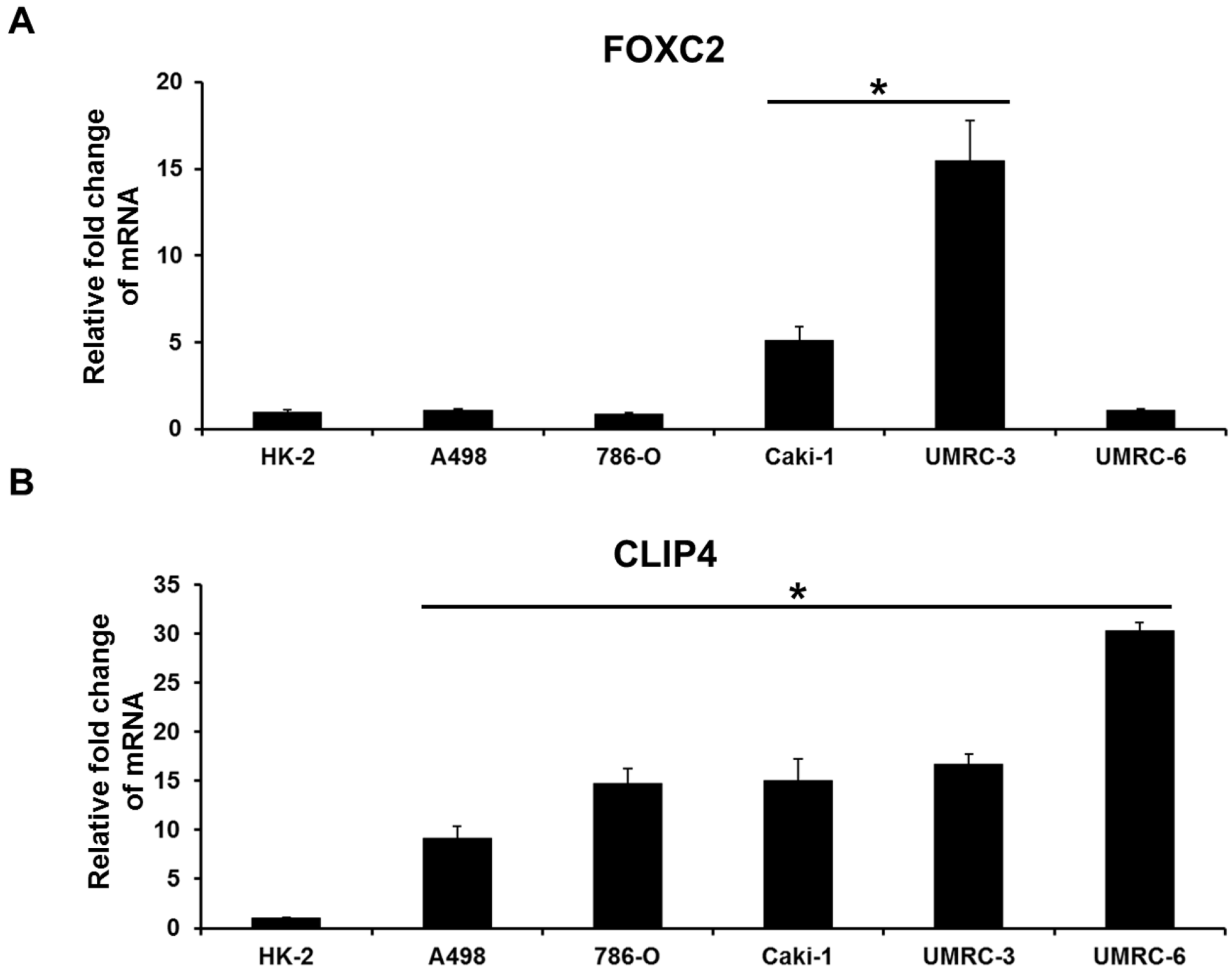

Supplementary Figure S1: Expression of FOXC2 and CLIP4 mRNA in RCC cell lines. Expression of *FOXC2* **A.** and *CLIP4* **B.** mRNA in normal renal tubular cells (HK-2) and a panel of RCC cell lines (A498, 786-O, Caki-1, UMRC-3, and UMRC-6). *, $p < 0.05$.

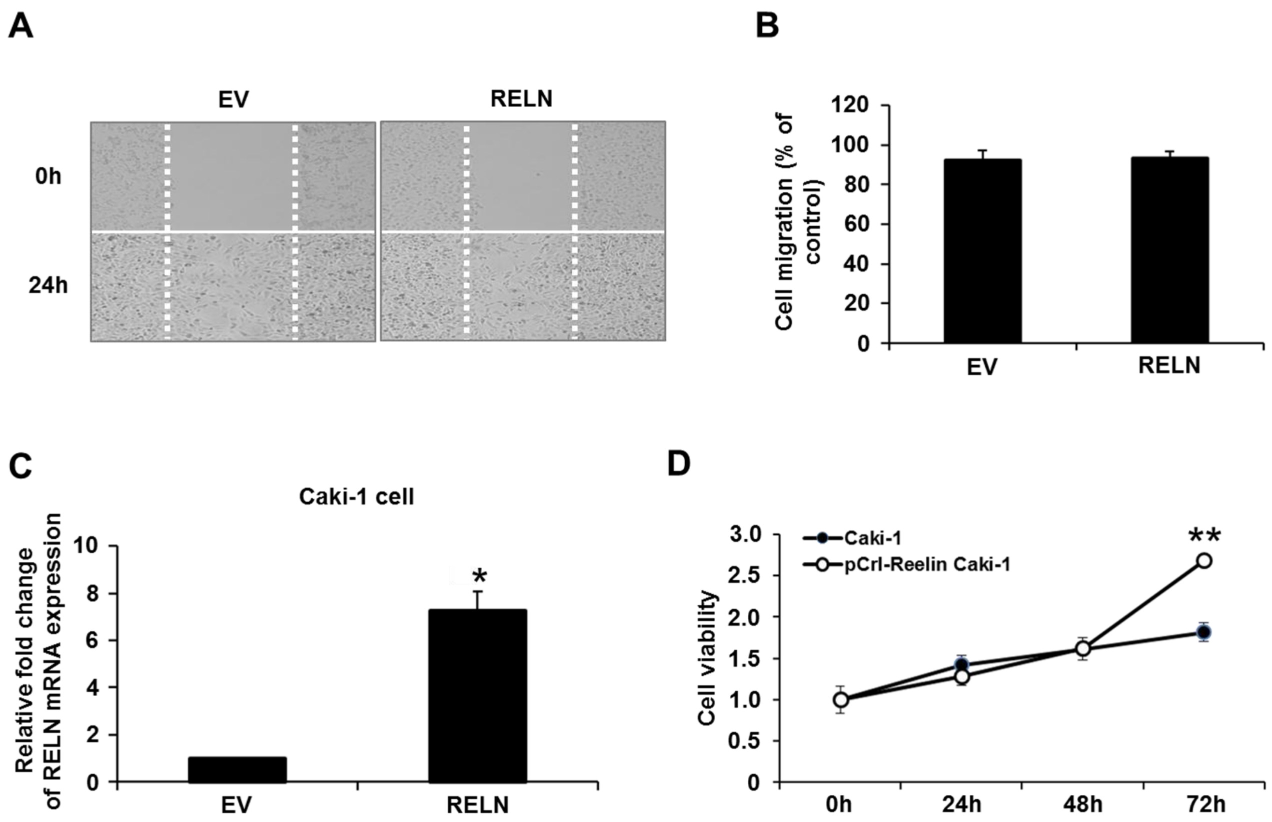

Supplementary Figure S2: In vitro cell migration and viability assay of RELN with gene overexpression. A. Representative image of cell migration in the wound-healing assay after *RELN* overexpression. **B.** Relative cell migration ratios are presented as the mean ± sd (n = 3). **C.** Relative fold change of *RELN* mRNA expression (n = 3; *, $p < 0.05$). **D.** Changes in cell viability over time following *RELN* overexpression (n = 3; **, $p < 0.01$).

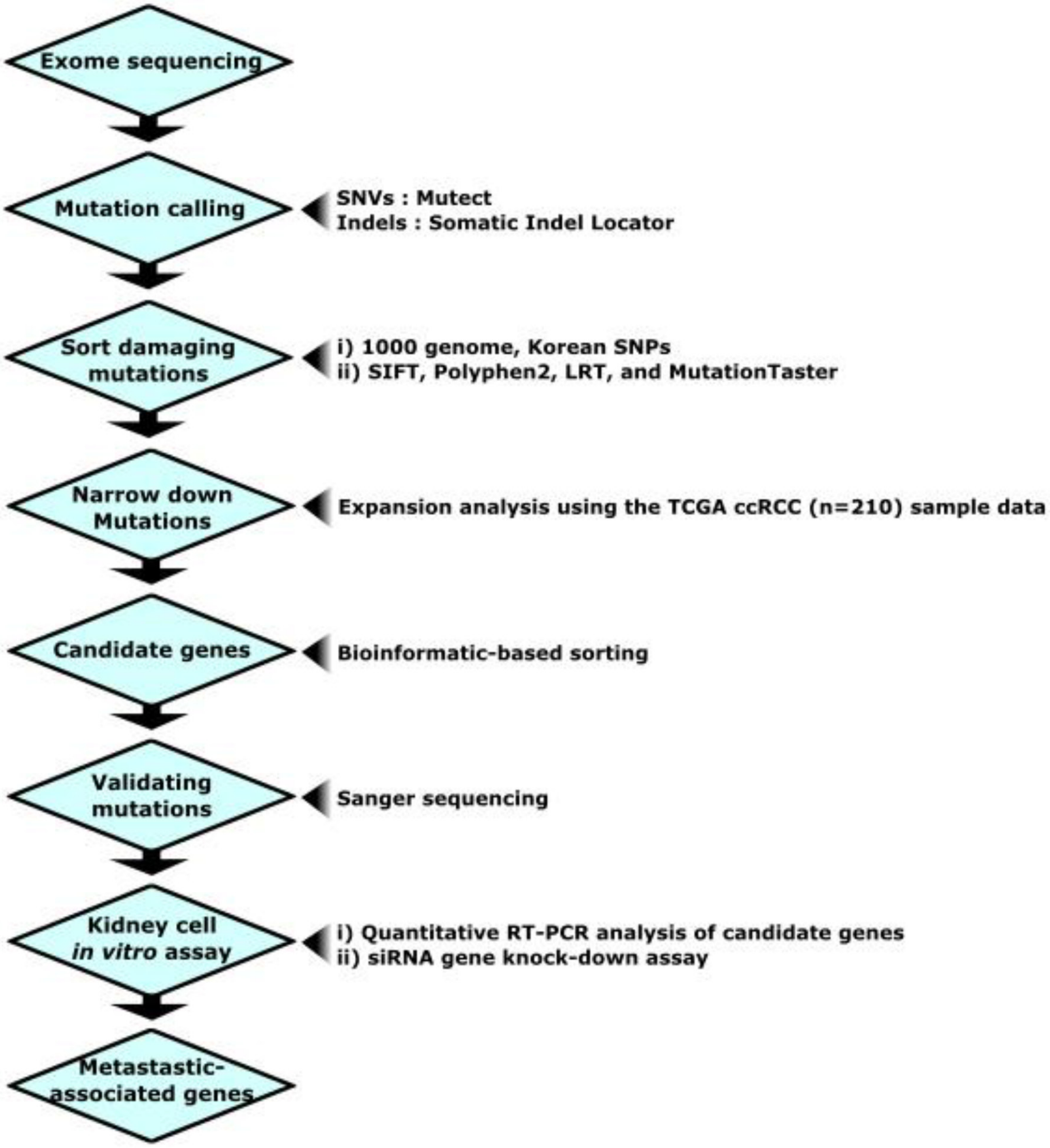

Supplementary Figure S3: Analysis flow pipeline. SNV, somatic nucleotide variant; SNP, single nucleotide polymorphism; LRT, likelihood ratio test; SIFT, sorting intolerant from tolerant]; TCGA, The Cancer Genome Atlas.

Supplementary Table S1: Clinical information for the 10 ccRCC samples with synchronous metastasis of ≤7-cm clear cell renal cell carcinomas

Sample ID	Gender	Age	Histology	Race	Stage	Diameter (mm)	Metastasis
RCC1	female	54	renal cell carcinoma, clear cell, Fuhrman grade 3	Asian	pT1a	20	lung, bone
RCC2	male	72	renal cell carcinoma, clear cell, Fuhrman grade 3	Asian	pT1a	20	chest wall
RCC3	male	58	renal cell carcinoma, clear cell, Fuhrman grade 2	Asian	pT1a	22	lung
RCC4	male	51	renal cell carcinoma, clear cell, Fuhrman grade 3	Asian	pT3a	27	bone
RCC5	female	51	renal cell carcinoma, clear cell, Fuhrman grade 3	Asian	pT1b	55	lymph node
RCC6	male	67	renal cell carcinoma, clear cell, Fuhrman grade 2	Asian	pT1a	24	bone
RCC7	male	45	renal cell carcinoma, clear cell, Fuhrman grade 3	Asian	pT1a	35	bone
RCC8	male	53	renal cell carcinoma, clear cell, Fuhrman grade 3	Asian	pT3a	65	bone
RCC9	female	67	renal cell carcinoma, clear cell, Fuhrman grade 3	Asian	pT1a	29	bone
RCC10	male	60	renal cell carcinoma, clear cell, Fuhrman grade 3	Asian	pT3b	45	lung

Supplementary Table S2: Summary of depth and coverage for whole exome sequencing data

Sample ID	Depth (×)	On target (%)	1 × (%)	5 × (%)	10 × (%)	20 × (%)	30 × (%)
RCC-T1	83.29	73.81	98.99	98.12	96.51	91.01	83.37
RCC-T2	77.70	73.85	98.94	97.59	95.11	87.55	78.49
RCC-T3	73.16	72.80	99.03	98.00	96.12	89.79	80.92
RCC-T4	90.14	70.18	99.19	98.43	97.07	92.47	85.71
RCC-T5	69.22	72.80	98.85	97.41	94.83	86.92	77.24
RCC-T6	89.49	70.65	99.01	97.98	96.36	91.68	85.28
RCC-T7	97.05	71.39	99.16	98.46	97.31	93.63	88.14
RCC-T8	92.03	70.42	99.12	98.30	96.95	92.77	86.76
RCC-T9	77.70	71.76	99.08	98.37	96.89	91.42	83.15
RCC-T10	67.23	71.10	99.11	98.06	95.87	88.20	77.49
RCC-N1	42.53	71.64	98.73	96.41	91.26	76.49	60.53
RCC-N2	45.23	44.58	98.57	94.92	88.18	72.45	57.27
RCC-N3	47.23	70.28	98.98	97.12	92.87	80.38	66.41
RCC-N4	55.91	68.27	99.11	97.80	94.92	85.25	73.00
RCC-N5	48.82	69.61	98.82	96.19	90.71	77.77	64.35
RCC-N6	55.06	69.02	99.04	97.64	94.70	84.85	72.54
RCC-N7	50.78	70.28	98.99	97.46	94.26	83.36	69.55
RCC-N8	57.25	69.98	99.10	97.88	95.11	85.96	74.50
RCC-N9	78.44	71.20	99.08	98.37	96.99	91.80	84.16
RCC-N10	99.22	69.37	99.24	98.67	97.74	94.59	89.62

Supplementary Table S3: Validation results using Sanger sequencing.

See Supplementary File 1

Supplementary Table S4: Total mutation information from the 10 ccRCC whole exome sequenced patients.

See Supplementary File 2

Supplementary Table S5: Somatic mutations in the 10 ccRCC whole exome sequenced patients

	Non-synonymous SNVs	Nonsense SNVs	Non-frameshift indels	Frameshift indels	Mutation/Mb
RCC1	15	1	2	0	0.60
RCC2	36	2	0	0	1.27
RCC3	21	0	0	1	0.73
RCC4	21	1	0	3	0.83
RCC5	7	0	0	0	0.23
RCC6	15	3	1	0	0.63
RCC7	4	0	0	0	0.13
RCC8	23	6	0	1	1.00
RCC9	26	4	0	0	1.00
RCC10	22	2	0	1	0.83

SNV, somatic nucleotide variant.

Supplementary Table S6: Mutation information for the frequently mutated candidate metastasis-associated genes.

See Supplementary File 3

Supplementary Table S7: Clinical information for the TCGA ccRCC patients used in expanded analysis.

See Supplementary File 4

REFERENCES

1. From ‘http://novocraft.com’ homepage.
2. Li H, Handsaker B, Wysoker A, Fennell T, Ruan J, Homer N, Marth G, Abecasis G, Durbin R. The Sequence Alignment/Map format and SAMtools. Bioinformatics. 2009; 25: 2078-2079.
3. From ‘http://picard.sourceforge.net’ homepage.
4. Cibulskis K, Lawrence MS, Carter SL, Sivachenko A, Jaffe D, Sougnez C, Gabriel S, Meyerson M, Lander ES, Getz G. Sensitive detection of somatic point mutations in impure and heterogeneous cancer samples. Nature biotechnology. 2013; 31: 213-219.
5. DePristo MA, Banks E, Poplin R, Garimella KV, Maguire JR, Hartl C, Philippakis AA, del Angel G, Rivas MA, Hanna M, McKenna A, Fennell TJ, Kernytsky AM, et al. A framework for variation discovery and genotyping using next-generation DNA sequencing data. Nature genetics. 2011; 43: 491-498.
6. Wang K, Li M, Hakonarson H. ANNOVAR: functional annotation of genetic variants from high-throughput sequencing data. Nucleic acids research. 2010; 38: e164.
7. Ng PC, Henikoff S. SIFT: Predicting amino acid changes that affect protein function. Nucleic acids research. 2003; 31: 3812-3814.
8. Adzhubei IA, Schmidt S, Peshkin L, Ramensky VE, Gerasimova A, Bork P, Kondrashov AS, Sunyaev SR. A method and server for predicting damaging missense mutations. Nature methods. 2010; 7: 248-249.
9. S. Chun, J. C. Fay. Identification of deleterious mutations within three human genomes. Genome Research. 2009; 19: 1553-1561.
10. Schwarz JM, Rödelsperger C, Schuelke M, Seelow D. MutationTaster evaluates disease-causing potential of sequence alterations. Nature methods. 2010; 7: 575-576.
