# Supplementary material for: FOXC2 and CLIP4 : a potential biomarker for synchronous metastasis of ≤7-cm clear cell renal cell carcinomas
Source: Oncotarget. 2016 Jun 6;7(32):51423–34. doi: 10.18632/oncotarget.9842 (PMC5239485; doi:10.18632/oncotarget.9842)
Supplement: Supplementary file 2 [file oncotarget-07-51423-s002.docx]

| **Supplementary Table S3: Validation results using Sanger sequencing.** | | | | | |
| --- | --- | --- | --- | --- | --- |
| **Gene** | **Position** | **Amino Acid Change** | **Forward Primer** | **Reverse Primer** | **Validation** |
| CLIP4 | chr2:29366783 | G286E | CTGCTAAGGAAATCAAGCAGATG | TACTGTGTCCTTGAACATAGGTC | Positive |
| CLIP4 | chr2:29380112 | S433C | CTGTTACAGTTGCCCTGCTT | GCACTGGCTCTGGATGAAAG | Positive |
| RELN | chr7:103193988 | F1998V | TAGAACAAAGTCAAGTACCTCAAAT | TCCTGGTGGTAACATCGGTC | Positive |
| ASAP1 | chr8:131191573 | I228S | CTACTCACTTGCACTGTGCA | AGGCTTGGTAACTTGTGCAG | Positive |
| CDH22 | chr20:44845551 | A251E | GTCATTGACGTCGGTGACTA | TCACCCTCACTCACCCACA | Positive |
| FOXC2 | chr16:86601630 | T230R | GTGTCCAAGGAGAAGGAGGA | TCATGATGTTCTCCACGCTG | Positive |
| MEF2A | chr15:100252737 | Q351 Nfs del | CATCAGCATCAAGTCCGAAC | CTAGAGCTGCTCAGACTGTC | Positive |
| HN1L | chr16:1747826 | L117X | GTTATATGGTCCTGATTTTCATTGTCC | AGACTAGCAACTCTTCCAGAA | Positive |
| INSRR | chr1:156810765 | C1265Y | CCATTTTGAGGGCTGCAGTC | CCATCTTTCACACACATTCTG | Positive |
| CNTNAP5 | chr2:125669095 | S1235N | CCAGTGCTTTGAGTGAGAAG | TAAGGAAAGCTTCAGATAACTTTCTCC | Positive |
| SYNC | chr1:33161206 | E165X | TGGATGAGCTGATCCCTCTC | CCAGAGCACGTTGTTTATGAG | Positive |
| DCAF8L2 | chrX:27766652 | A547V | GGTACAATAAACTGTCTTGAACC | AAGCATGTACTGGTCAAACAGG | Positive |
| ZFR | chr5:32406959 | Q318X | CATACAACGTAAGTACCTGTGG | CAGCAACAGAAGCAGGCA | Positive |
| ANGPT1 | chr8:108264210 | L457P | GTTGTGGAACGTAAGGAGTAAC | GTTGCCTCTCCTTCTCTCTTC | Positive |
| PLCE1 | chr10:96014739 | G1163R | CATCCTCACAGAGGTGAATG | GGGTCCTTCTGTTTTATCATGTT | Positive |
| INSRR | chr1:156812881 | T1014M | TTGGCTCTCCCTTACCACAT | GCTCTTTTGGGATGGTATATG | Negative |
| MLL2 | chr12:49416603 | G5370S | GGTACACGTTGTTCTTCCATTC | GGAGAGCTGTCTTGTCATGG | Positive |
| DIP2A | chr21:47931351 | Q266R | CAGAATGTGTTCGTGGTGCT | CAAGGTGGCCAACAGCGAC | Positive |
| RIMBP2 | chr12:130897283 | A901G | CTGTCTAAGAAGCTGATCCATC | CTGAACCAATCAATTACAGTGC | Positive |
| ZFC3H1 | chr12:72050806 | Q292E | CGGTTCTTATCTCCTGGTAAAG | CCTAAAACATTGAACTTCGAGG | Positive |
