# Supplementary material for: FOXC2 and CLIP4 : a potential biomarker for synchronous metastasis of ≤7-cm clear cell renal cell carcinomas
Source: Oncotarget. 2016 Jun 6;7(32):51423–34. doi: 10.18632/oncotarget.9842 (PMC5239485; doi:10.18632/oncotarget.9842)
Supplement: Supplementary file 3 [file oncotarget-07-51423-s003.docx]

**Table S4. Total mutation information from the 10 ccRCC whole exome sequenced patients.**

| **Gene** | **Position** | **Chromosome** | **Position** | **Ref allele** | **Alt Allele** | **Amino Acid Change** | **Sample** |
| --- | --- | --- | --- | --- | --- | --- | --- |
| *LY9* | Missense | 1 | 160783642 | C | G | A224G | RCC1 |
| *CLIP4* | Missense | 2 | 29366783 | G | A | G286E | RCC1 |
| *TTN* | Missense | 2 | 179431555 | T | G | K17370T | RCC1 |
| *VHL* | Nonsense | 3 | 10191524 | G | T | E173X | RCC1 |
| *TRIM31* | Missense | 6 | 30078297 | C | A | Q224H | RCC1 |
| *ANKS1A* | Missense | 6 | 35047435 | A | G | E809G | RCC1 |
| *SYNJ2* | Missense | 6 | 158516750 | G | A | R1282Q | RCC1 |
| *ANGPT1* | Missense | 8 | 108264210 | A | G | L457P | RCC1 |
| *TBX10* | Missense | 11 | 67402605 | G | A | A46V | RCC1 |
| *THSD1* | Missense | 13 | 52952141 | C | G | S602T | RCC1 |
| *EXD2* | Missense | 14 | 69695776 | G | A | A193T | RCC1 |
| *ACAN* | Missense | 15 | 89401074 | C | A | T1753N | RCC1 |
| *LIN37* | Missense | 19 | 36245350 | T | C | L239P | RCC1 |
| *SLC13A3* | Missense | 20 | 45221109 | A | C | L238R | RCC1 |
| *GTSE1* | Missense | 22 | 46693361 | G | A | D22N | RCC1 |
| *DCAF8L2* | Missense | X | 27766652 | C | T | A547V | RCC1 |
| *KAT6A* | Non-Frameshift Indel | 8 | 41794804 | CTTC | C | 3319_3322 Nfs del | RCC1 |
| *MEGF9* | Non-Frameshift Indel | 9 | 123476562 | GGCGGCG | G | 69_75 Nfs del | RCC1 |
| *TNN* | Missense | 1 | 175067515 | A | G | M635V | RCC2 |
| *LAD1* | Missense | 1 | 201352467 | T | G | E409A | RCC2 |
| *FAM168B* | Missense | 2 | 131829467 | G | A | P39S | RCC2 |
| *VHL* | Missense | 3 | 10191530 | T | G | Y175D | RCC2 |
| *NT5DC2* | Missense | 3 | 52559238 | T | C | Y398C | RCC2 |
| *PHF17* | Missense | 4 | 129783018 | G | T | G381W | RCC2 |
| *TNXB* | Missense | 6 | 32015692 | T | G | E3379D | RCC2 |
| *HOXA13* | Missense | 7 | 27238939 | G | C | P253R | RCC2 |
| *FKBP9* | Missense | 7 | 33044951 | C | G | H567Q | RCC2 |
| *UBE3C* | Missense | 7 | 157013416 | G | A | V650M | RCC2 |
| *ASPH* | Missense | 8 | 62578006 | C | G | G161A | RCC2 |
| *NR5A1* | Missense | 9 | 127262475 | C | T | R255H | RCC2 |
| *SETX* | Missense | 9 | 135211891 | C | A | W170C | RCC2 |
| *TUBB4B* | Missense | 9 | 140136979 | G | T | K103N | RCC2 |
| *GBF1* | Missense | 10 | 104119126 | T | C | S372P | RCC2 |
| *FXC1* | Missense | 11 | 6503024 | A | C | E26A | RCC2 |
| *LRRC4C* | Missense | 11 | 40137749 | C | A | A32S | RCC2 |
| *SLC35C1* | Nonsense | 11 | 45827443 | G | T | E18X | RCC2 |
| *ARHGEF17* | Missense | 11 | 73071447 | C | T | S1430F | RCC2 |
| *C11orf30* | Missense | 11 | 76255787 | C | A | P1065Q | RCC2 |
| *MRPL51* | Nonsense | 12 | 6602073 | C | A | E49X | RCC2 |
| *MLL2* | Missense | 12 | 49416603 | C | T | G5370S | RCC2 |
| *BAZ2A* | Missense | 12 | 56995538 | G | T | P1290H | RCC2 |
| *TUBA3C* | Missense | 13 | 19751243 | C | A | A294S | RCC2 |
| *KLHL28* | Missense | 14 | 45398308 | C | G | D547H | RCC2 |
| *LIN52* | Missense | 14 | 74564563 | G | C | E71Q | RCC2 |
| *FEM1B* | Missense | 15 | 68582559 | A | T | D288V | RCC2 |
| *CHD2* | Missense | 15 | 93543782 | A | G | N1350S | RCC2 |
| *CLCN7* | Missense | 16 | 1498685 | G | A | A627V | RCC2 |
| *FOXC2* | Missense | 16 | 86601630 | C | G | T230R | RCC2 |
| *SMG6* | Missense | 17 | 2202881 | G | A | S358F | RCC2 |
| *TRIM65* | Missense | 17 | 73888507 | G | T | H195Q | RCC2 |
| *ELL* | Missense | 19 | 18561653 | G | A | P367S | RCC2 |
| *EHD2* | Missense | 19 | 48229071 | T | A | Y169N | RCC2 |
| *SIGLEC7* | Missense | 19 | 51650095 | T | C | V278A | RCC2 |
| *DSCAM* | Missense | 21 | 41414391 | C | T | E1865K | RCC2 |
| *ZNF280A* | Missense | 22 | 22869780 | C | T | V59I | RCC2 |
| *BCORL1* | Missense | X | 129146942 | T | C | V65A | RCC2 |
| *KIAA1751* | Missense | 1 | 1918446 | G | A | R109W | RCC3 |
| *MPL* | Missense | 1 | 43815014 | T | C | F517L | RCC3 |
| *NBPF14* | Missense | 1 | 148011027 | C | T | G532E | RCC3 |
| *IL10* | Missense | 1 | 206944260 | G | A | R124W | RCC3 |
| *YOD1* | Missense | 1 | 207222520 | G | C | L298V | RCC3 |
| *CNTNAP5* | Missense | 2 | 125669095 | G | A | S1235N | RCC3 |
| *UGT1A4* | Missense | 2 | 234627746 | C | A | L94M | RCC3 |
| *CDO1* | Missense | 5 | 115152045 | C | T | R17H | RCC3 |
| *SCGB3A2* | Missense | 5 | 147261131 | G | C | V60L | RCC3 |
| *LAMA2* | Missense | 6 | 129513853 | C | A | T546N | RCC3 |
| *RELN* | Missense | 7 | 103193988 | A | C | F1998V | RCC3 |
| *OR56A4* | Missense | 11 | 6023655 | G | A | L242F | RCC3 |

| **Gene** | **Position** | **Chromosome** | **Position** | **Ref allele** | **Alt Allele** | **Amino Acid Change** | **Sample** |
| --- | --- | --- | --- | --- | --- | --- | --- |
| *PEX5* | Missense | 12 | 7362334 | A | C | E502A | RCC3 |
| *RASSF9* | Missense | 12 | 86199420 | A | G | V123A | RCC3 |
| *PCDH8* | Missense | 13 | 53420525 | C | A | G683C | RCC3 |
| *RIN3* | Missense | 14 | 93151439 | C | T | R859C | RCC3 |
| *KIF26A* | Missense | 14 | 104638060 | G | A | V372M | RCC3 |
| *ATMIN* | Missense | 16 | 81077894 | C | G | I597M | RCC3 |
| *MAP2K7* | Missense | 19 | 7975221 | T | C | M137T | RCC3 |
| *KLK14* | Missense | 19 | 51581380 | G | A | L230F | RCC3 |
| *SIGLEC1* | Missense | 20 | 3677280 | G | A | T879I | RCC3 |
| *PBRM1* | Frameshift Indel | 3 | 52692318 | TA | T | 541_542 fs del | RCC3 |
| *CAD* | Missense | 2 | 27457011 | C | G | L1179V | RCC4 |
| *CYP20A1* | Missense | 2 | 204161228 | G | T | V394L | RCC4 |
| *BCHE* | Missense | 3 | 165491253 | G | A | H576Y | RCC4 |
| *OCIAD2* | Missense | 4 | 48894849 | C | G | S108T | RCC4 |
| *MAB21L2* | Missense | 4 | 151505071 | G | A | G297D | RCC4 |
| *SCRN1* | Missense | 7 | 29994961 | T | C | I59V | RCC4 |
| *AZGP1* | Missense | 7 | 99569609 | T | C | I33V | RCC4 |
| *PLCE1* | Missense | 10 | 96014739 | G | A | G1163R | RCC4 |
| *CDC42BPG* | Missense | 11 | 64597447 | C | T | V1155M | RCC4 |
| *GRIN2B* | Missense | 12 | 13716891 | G | C | A1094G | RCC4 |
| *UNG* | Missense | 12 | 109536384 | T | A | F85I | RCC4 |
| *SCAMP5* | Missense | 15 | 75311157 | G | C | G181R | RCC4 |
| *SPSB3* | Nonsense | 16 | 1827329 | G | T | Y279X | RCC4 |
| *EIF4A1* | Missense | 17 | 7479906 | G | C | G137A | RCC4 |
| *DUS1L* | Missense | 17 | 80017869 | T | G | K375Q | RCC4 |
| *ZNF266* | Missense | 19 | 9524577 | G | T | H342N | RCC4 |
| *ZNF536* | Missense | 19 | 30935785 | T | G | M439R | RCC4 |
| *RALGAPA2* | Missense | 20 | 20601134 | T | A | L458F | RCC4 |
| *CDH22* | Missense | 20 | 44845551 | G | T | A251E | RCC4 |
| *CACNA1F* | Missense | X | 49071643 | G | T | P1178Q | RCC4 |
| *GDPD2* | Missense | X | 69649395 | C | T | A330V | RCC4 |
| *KIAA2022* | Missense | X | 73960554 | A | G | S1280P | RCC4 |
| *PYCR2* | Frameshift Indel | 1 | 226109636 | GC | G | 461_462 fs del | RCC4 |
| *UBE4A* | Frameshift Indel | 11 | 118244323 | GA | G | 1060_1061 fs del | RCC4 |
| *KIF7* | Frameshift Indel | 15 | 90190137 | GC | G | 1711_1712 fs del | RCC4 |
| *TMEM37* | Missense | 2 | 120194617 | C | A | F58L | RCC5 |
| *SHQ1* | Missense | 3 | 72861887 | G | A | P332L | RCC5 |
| *METTL2B* | Missense | 7 | 128119380 | G | C | C124S | RCC5 |
| *OR5D16* | Missense | 11 | 55606377 | C | G | I50M | RCC5 |
| *SLC43A3* | Missense | 11 | 57175355 | G | C | F462L | RCC5 |
| *RAE1* | Missense | 20 | 55948631 | C | T | P248L | RCC5 |
| *ZNF831* | Missense | 20 | 57767479 | C | A | P469T | RCC5 |
| *KLHDC7A* | Missense | 1 | 18808652 | G | T | G393C | RCC6 |
| *SNTG2* | Missense | 2 | 1161313 | T | A | L164H | RCC6 |
| *DBR1* | Missense | 3 | 137881171 | G | T | Q399K | RCC6 |
| *ITPR3* | Missense | 6 | 33589426 | T | C | L16P | RCC6 |
| *STXBP5* | Missense | 6 | 147556393 | C | T | R86C | RCC6 |
| *TRIM32* | Missense | 9 | 119461682 | C | T | S554F | RCC6 |
| *ANK3* | Nonsense | 10 | 61830954 | C | A | E3229X | RCC6 |
| *HSPA8* | Missense | 11 | 122931860 | T | C | Q58R | RCC6 |
| *CSAD* | Missense | 12 | 53565219 | G | A | S180F | RCC6 |
| *TEX9* | Nonsense | 15 | 56657816 | C | T | Q23X | RCC6 |
| *LRRK1* | Missense | 15 | 101566239 | G | T | V768L | RCC6 |
| *GDE1* | Nonsense | 16 | 19516328 | A | T | Y241X | RCC6 |
| *HYDIN* | Missense | 16 | 70871601 | T | C | K4411E | RCC6 |
| *TLE6* | Missense | 19 | 2991887 | G | A | G431S | RCC6 |
| *C20orf26* | Missense | 20 | 20056186 | T | C | Y165H | RCC6 |
| *IL10RB* | Missense | 21 | 34652176 | T | A | S151T | RCC6 |
| *DIP2A* | Missense | 21 | 47931351 | A | G | Q266R | RCC6 |
| *PLXNB2* | Missense | 22 | 50716631 | C | A | G1601V | RCC6 |
| *PTPRZ1* | Non-Frameshift Indel | 7 | 121653389 | ATGA | A | 4289_4292 Nfs del | RCC6 |
| *CLIP4* | Missense | 2 | 29380112 | C | G | S433C | RCC7 |
| *PLXDC2* | Missense | 10 | 20568704 | C | A | P516T | RCC7 |
| *KIAA1704* | Missense | 13 | 45589567 | G | A | D181N | RCC7 |
| *PPP2R1A* | Missense | 19 | 52714657 | G | A | D139N | RCC7 |
| *IQGAP3* | Missense | 1 | 156508752 | G | A | R1044C | RCC8 |
| *INSRR* | Missense | 1 | 156812881 | G | A | T1014M | RCC8 |
| *ETV3* | Missense | 1 | 157094773 | C | G | D467H | RCC8 |
| *SIPA1L2* | Missense | 1 | 232607237 | G | A | T708I | RCC8 |

| **Gene** | **Position** | **Chromosome** | **Position** | **Ref allele** | **Alt Allele** | **Amino Acid Change** | **Sample** |
| --- | --- | --- | --- | --- | --- | --- | --- |
| *KBTBD10* | Nonsense | 2 | 170366466 | G | T | E60X | RCC8 |
| *VHL* | Nonsense | 3 | 10183817 | C | T | Q96X | RCC8 |
| *KCNH8* | Missense | 3 | 19575368 | T | C | V1034A | RCC8 |
| *TMPRSS7* | Missense | 3 | 111780677 | C | T | P326S | RCC8 |
| *VWA5B2* | Nonsense | 3 | 183956239 | G | A | W623X | RCC8 |
| *ODZ3* | Missense | 4 | 183245337 | C | T | S55L | RCC8 |
| *SLC9A3* | Missense | 5 | 476162 | C | T | A705T | RCC8 |
| *ZFR* | Nonsense | 5 | 32406959 | G | A | Q318X | RCC8 |
| *GABRA6* | Missense | 5 | 161113247 | C | G | A17G | RCC8 |
| *NSD1* | Missense | 5 | 176722263 | T | C | S2363P | RCC8 |
| *HLA-F* | Missense | 6 | 29692825 | G | A | V210I | RCC8 |
| *MTUS1* | Nonsense | 8 | 17612230 | G | A | Q363X | RCC8 |
| *TRPM6* | Missense | 9 | 77397737 | C | A | M979I | RCC8 |
| *EGR2* | Missense | 10 | 64573097 | G | A | S384L | RCC8 |
| *TRIM8* | Nonsense | 10 | 104404927 | C | T | R185X | RCC8 |
| *MUC2* | Missense | 11 | 1093016 | C | A | T1612N | RCC8 |
| *TMEM117* | Missense | 12 | 44238597 | G | T | G48V | RCC8 |
| *ZNF410* | Missense | 14 | 74364868 | C | A | S178R | RCC8 |
| *SPG11* | Missense | 15 | 44951411 | A | T | F178Y | RCC8 |
| *COX6A2* | Missense | 16 | 31439106 | C | T | E95K | RCC8 |
| *COX6A2* | Missense | 16 | 31439385 | C | A | R54L | RCC8 |
| *COMP* | Missense | 19 | 18895860 | T | C | H587R | RCC8 |
| *ZSWIM3* | Missense | 20 | 44506253 | G | T | Q352H | RCC8 |
| *DIDO1* | Missense | 20 | 61541267 | G | C | I315M | RCC8 |
| *RBMXL3* | Missense | X | 114426712 | G | A | R903Q | RCC8 |
| *FAM98A* | Frameshift Indel | 2 | 33810195 | GA | G | 1204_1205 fs del | RCC8 |
| *LOC440563* | Missense | 1 | 13183833 | C | T | V14M | RCC9 |
| *SYNC* | Nonsense | 1 | 33161206 | C | A | E165X | RCC9 |
| *RIT1* | Missense | 1 | 155874189 | A | C | I114M | RCC9 |
| *ARHGEF11* | Missense | 1 | 156907180 | G | T | T1434K | RCC9 |
| *OR6P1* | Missense | 1 | 158532706 | G | A | T230M | RCC9 |
| *RXRG* | Missense | 1 | 165370548 | A | C | I325M | RCC9 |
| *ASAP2* | Missense | 2 | 9437459 | A | G | Q77R | RCC9 |
| *NBAS* | Missense | 2 | 15467940 | C | T | V1506I | RCC9 |
| *MSH6* | Nonsense | 2 | 48030654 | G | T | E1090X | RCC9 |
| *LY75-CD302* | Missense | 2 | 160735774 | T | C | D504G | RCC9 |
| *VHL* | Missense | 3 | 10183776 | G | C | R82P | RCC9 |
| *PBRM1* | Missense | 3 | 52651518 | T | A | L526F | RCC9 |
| *CACNA2D3* | Missense | 3 | 54905585 | G | A | R549Q | RCC9 |
| *HNRNPAB* | Missense | 5 | 177636445 | G | A | G262R | RCC9 |
| *TNF* | Missense | 6 | 31545225 | G | A | G205S | RCC9 |
| *ANKRD6* | Missense | 6 | 90326279 | C | T | A173V | RCC9 |
| *KIAA1919* | Missense | 6 | 111587721 | C | T | P319L | RCC9 |
| *MLLT4* | Missense | 6 | 168363164 | C | T | R1620W | RCC9 |
| *ASAP1* | Missense | 8 | 131191573 | A | C | I228S | RCC9 |
| *KRT80* | Missense | 12 | 52574704 | A | T | F185I | RCC9 |
| *TMPO* | Missense | 12 | 98931261 | A | G | I192V | RCC9 |
| *PLEKHG3* | Missense | 14 | 65198183 | G | T | R262S | RCC9 |
| *C15orf48* | Missense | 15 | 45723275 | C | T | T38I | RCC9 |
| *HN1L* | Nonsense | 16 | 1747826 | T | A | L117X | RCC9 |
| *RBBP6* | Missense | 16 | 24582865 | A | C | E1459A | RCC9 |
| *CDT1* | Missense | 16 | 88871064 | G | A | A114T | RCC9 |
| *PSMD3* | Missense | 17 | 38151740 | A | G | Q427R | RCC9 |
| *EVI5L* | Missense | 19 | 7928559 | G | A | A786T | RCC9 |
| *ZNF625* | Nonsense | 19 | 12256780 | G | A | R151X | RCC9 |
| *CYB5R3* | Missense | 22 | 43040442 | C | T | R25H | RCC9 |
| *PTPRU* | Missense | 1 | 29581882 | C | A | H57N | RCC10 |
| *EXO1* | Missense | 1 | 242042300 | T | G | F588L | RCC10 |
| *LTBP1* | Missense | 2 | 33585825 | C | A | P967T | RCC10 |
| *POLR1A* | Missense | 2 | 86315782 | A | C | S213A | RCC10 |
| *MME* | Missense | 3 | 154855951 | A | G | R261G | RCC10 |
| *HAUS3* | Missense | 4 | 2242273 | G | A | S134F | RCC10 |
| *SSBP2* | Missense | 5 | 80946145 | C | T | V26I | RCC10 |
| *SQSTM1* | Missense | 5 | 179260094 | C | T | P273S | RCC10 |
| *BMP5* | Missense | 6 | 55739378 | C | T | E96K | RCC10 |
| *MBLAC1* | Missense | 7 | 99725459 | C | A | H147Q | RCC10 |
| *FAM208B* | Missense | 10 | 5791427 | A | T | I2015F | RCC10 |
| *SMC3* | Missense | 10 | 112333494 | T | C | F41L | RCC10 |
| **Gene** | **Position** | **Chromosome** | **Position** | **Ref allele** | **Alt Allele** | **Amino Acid Change** | **Sample** |
| *OR4A15* | Nonsense | 11 | 55136097 | T | A | Y246X | RCC10 |
| *FOXRED1* | Missense | 11 | 126143253 | C | T | A147V | RCC10 |
| *MLL2* | Missense | 12 | 49432561 | G | A | R2860C | RCC10 |
| *LGR5* | Missense | 12 | 71978477 | G | A | C896Y | RCC10 |
| *ZFC3H1* | Missense | 12 | 72050806 | G | C | Q292E | RCC10 |
| *RIMBP2* | Missense | 12 | 130897283 | G | C | A901G | RCC10 |
| *RFC3* | Missense | 13 | 34410400 | A | T | M347L | RCC10 |
| *ANKRD12* | Missense | 18 | 9257283 | G | A | G1317R | RCC10 |
| *ZNF521* | Missense | 18 | 22806432 | C | T | E484K | RCC10 |
| *CLPTM1* | Missense | 19 | 45493688 | G | A | G390S | RCC10 |
| *C2CD2* | Missense | 21 | 43319380 | G | A | P551L | RCC10 |
| *HDX* | Nonsense | X | 83581188 | G | A | Q649X | RCC10 |
| *LFNG* | Frameshift Indel | 7 | 2552905 | GGATG | G | 162_166 fs del | RCC10 |
