# Supplementary material for: FOXC2 and CLIP4 : a potential biomarker for synchronous metastasis of ≤7-cm clear cell renal cell carcinomas
Source: Oncotarget. 2016 Jun 6;7(32):51423–34. doi: 10.18632/oncotarget.9842 (PMC5239485; doi:10.18632/oncotarget.9842)
Supplement: Supplementary file 4 [file oncotarget-07-51423-s004.docx]

**Table S6. Mutation information for the frequently mutated candidate metastasis-associated genes.**

| **Gene** | **Mutation frequency in our set (n=10)** | **Mutation frequency in TCGA group A (n=5)** | **Mutation frequency  in TCGA group C (n=36)** | **Total (n)** | **AA substitution** | **No. of  damage algorithms** |
| --- | --- | --- | --- | --- | --- | --- |
| *ANGPT1* | 1/10 | - | 1/36 | 2/51 | L457P | 4/4 |
|  |  |  |  |  | E149G | 4/4 |
| *ANK3* | 1/10 | - | 2/36 | 3/51 | E3229X | 0/1 |
|  |  |  |  |  | R157P | 3/4 |
|  |  |  |  |  | S3712F | 2/3 |
| *ASAP1* | 1/10 | - | 1/36 | 2/51 | I228S | 3/4 |
|  |  |  |  |  | T393A | 3/4 |
| *ATMIN* | 1/10 | - | 1/36 | 2/51 | I597M | 4/4 |
|  |  |  |  |  | S500N | 0/4 |
| *BCHE* | 1/10 | - | 1/36 | 2/51 | H576Y | 0/4 |
|  |  |  |  |  | W550R | 4/4 |
| *CACNA1F* | 1/10 | - | 1/36 | 2/51 | P1178Q | 1/4 |
|  |  |  |  |  | T1509M | 3/4 |
| *CDH22* | 1/10 | - | 1/36 | 2/51 | A251E | 3/4 |
|  |  |  |  |  | V634I | 1/4 |
| *CDT1* | 1/10 | - | 1/36 | 2/51 | A114T | 0/4 |
|  |  |  |  |  | Y247D | 4/4 |
| *CLCN7* | 1/10 | - | 1/36 | 2/51 | A603V | 3/4 |
|  |  |  |  |  | S61N | 0/4 |
| *CLIP4* | 2/10 | 1/5 | - | 3/51 | G286E | 4/4 |
|  |  |  |  |  | S433C | 2/4 |
|  |  |  |  |  | G483E | 1/4 |
| *CNTNAP5* | 1/10 | - | 1/36 | 2/51 | S1235N | NA |
|  |  |  |  |  | F1063V | NA |
| *DCAF8L2* | 1/10 | - | 1/36 | 2/51 | A547V | NA |
|  |  |  |  |  | T588M | NA |
| *DIP2A* | 1/10 | - | 1/36 | 2/51 | Q245R | NA |
|  |  |  |  |  | R852H | NA |
| *DSCAM* | 1/10 | - | 1/36 | 2/51 | E1865K | 4/4 |
|  |  |  |  |  | T178M | 3/4 |
| *FAM98A* | 1/10 | - | 1/36 | 2/51 | 1204_1205 fs del | NA |
|  |  |  |  |  | L77I | 3/4 |
| *FOXC2* | 1/10 | - | 1/36 | 2/51 | T230R | 1/2 |
|  |  |  |  |  | G43D | 3/3 |
| *HN1L* | 1/10 | - | 1/36 | 2/51 | L117X | 1/2 |
|  |  |  |  |  | D161N | 1/4 |
| *HYDIN* | 1/10 | - | 2/36 | 3/51 | K4412E | 0/3 |
|  |  |  |  |  | M2133I | 0/1 |
|  |  |  |  |  | F2692L | 0/2 |
| *INSRR* | 1/10 | 1/5 | - | 2/51 | C1265Y | 1/4 |
|  |  |  |  |  | R1044H | 4/4 |
| *KIAA1751* | 1/10 | - | 1/36 | 2/51 | R109W | 1/4 |
|  |  |  |  |  | K97N | 2/4 |
| *KIAA2022* | 1/10 | - | 1/36 | 2/51 | S1280P | 1/4 |
|  |  |  |  |  | Q957H | 0/4 |
| *LTBP1* | 1/10 | - | 1/36 | 2/51 | P1020T | 1/2 |
|  |  |  |  |  | A469V | 1/3 |
| *MLL2* | 2/10 | - | 1/36 | 3/51 | G5370S | NA |
|  |  |  |  |  | R2860C | NA |
|  |  |  |  |  | C874X | NA |
|  |  |  |  |  |  |  |
|  |  |  |  |  |  |  |
| **Gene** | **Mutation frequency in our set (n=10)** | **Mutation frequency in TCGA group A (n=5)** | **Mutation frequency  in TCGA group C (n=36)** | **Total (n)** | **AA substitution** | **No. of  damage algorithms** |
| *MUC2* | 1/10 | - | 1/36 | 2/51 | T1612N | NA |
|  |  |  |  |  | T1756N | NA |
| *PLCE1* | 1/10 | - | 1/36 | 2/51 | G855R | 2/4 |
|  |  |  |  |  | 5281_5282 fs Ins | NA |
| *PTPRZ1* | 1/10 | - | 1/36 | 2/51 | 4289_4292 Nfs del | NA |
|  |  |  |  |  | G1136C | 1/3 |
| *RASSF9* | 1/10 | - | 1/36 | 2/51 | V123A | NA |
|  |  |  |  |  | E106X | NA |
| *RELN* | 1/10 | 1/5 | 2/36 | 4/51 | F1998V | 4/4 |
|  |  |  |  |  | A535P | 3/4 |
|  |  |  |  |  | P280T | 4/4 |
|  |  |  |  |  | R2428W | 4/4 |
| *RIMBP2* | 1/10 | - | 1/36 | 2/51 | A901G | 1/4 |
|  |  |  |  |  | E468K | 4/4 |
| *SCRN1* | 1/10 | - | 1/36 | 2/51 | I59V | 0/4 |
|  |  |  |  |  | D339Y | 4/4 |
| *SETX* | 1/10 | - | 1/36 | 2/51 | W170C | 4/4 |
|  |  |  |  |  | R2414Q | 3/4 |
| *SIPA1L2* | 1/10 | - | 1/36 | 2/51 | T708I | 4/4 |
|  |  |  |  |  | W903X | 2/2 |
| *SYNC* | 1/10 | - | 1/36 | 2/51 | E165X | NA |
|  |  |  |  |  | G480R | 4/4 |
| *TTN* | 1/10 | - | 1/36 | 2/51 | K17370T | NA |
|  |  |  |  |  | I23512F | NA |
|  |  |  |  |  | S2866F | 1/1 |
|  |  |  |  |  | P8180T | NA |
|  |  |  |  |  | A8529E | NA |
|  |  |  |  |  | I20227V | NA |
|  |  |  |  |  | G21134E | NA |
|  |  |  |  |  | Q4187R | 0/1 |
|  |  |  |  |  | E169D | 1/2 |
|  |  |  |  |  | M19159I | NA |
|  |  |  |  |  | D23757A | NA |
|  |  |  |  |  | I26395T | NA |
|  |  |  |  |  | I15363T | NA |
|  |  |  |  |  | C12512Y | NA |
|  |  |  |  |  | R6858S | NA |
|  |  |  |  |  | E4352A | NA |
|  |  |  |  |  | N21238K | NA |
|  |  |  |  |  | A3501S | NA |
|  |  |  |  |  | G8197R | NA |
|  |  |  |  |  | E10551X | NA |
|  |  |  |  |  | P6525T | NA |
|  |  |  |  |  | R3147X | NA |
|  |  |  |  |  | P14159L | NA |
| *ZFC3H1* | 1/10 | - | 1/36 | 2/51 | Q292E | 0/4 |
|  |  |  |  |  | R468G | 2/2 |
| *ZFR* | 1/10 | - | 1/36 | 2/51 | Q318X | 2/2 |
|  |  |  |  |  | C387S | 4/4 |
| *ZNF831* | 1/10 | - | 1/36 | 2/51 | P469T | 1/2 |
|  |  |  |  |  | 262_262 fs del | NA |
