# Supplementary material for: FOXC2 and CLIP4 : a potential biomarker for synchronous metastasis of ≤7-cm clear cell renal cell carcinomas
Source: Oncotarget. 2016 Jun 6;7(32):51423–34. doi: 10.18632/oncotarget.9842 (PMC5239485; doi:10.18632/oncotarget.9842)
Supplement: Supplementary file 5 [file oncotarget-07-51423-s005.docx]

**Table S7. Clinical information for the TCGA ccRCC patients used in expanded analysis.**

| **Sorted Group** | **Patient barcode** | **Sex** | **Age at diagnosis** | **Histological type** | **Race** | **Stage** | **Pathologic_N** | **Pathologic_M** |
| --- | --- | --- | --- | --- | --- | --- | --- | --- |
| Group A | TCGA-BP-4970 | MALE | 44 | RCC | ASIAN | T1a | N1 | M0 |
| Group A | TCGA-B0-5092 | FEMALE | 53 | ccRCC | WHITE | T1a | N0 | M1 |
| Group A | TCGA-A3-3347 | FEMALE | 76 | ccRCC | WHITE | T1b | N1 | M0 |
| Group A | TCGA-CZ-5461 | MALE | 52 | ccRCC | WHITE | T1b | NX | M1 |
| Group A | TCGA-CZ-5462 | MALE | 83 | ccRCC | WHITE | T1b | NX | M1 |
| Group B | TCGA-CZ-5986 | MALE | 61 | ccRCC | WHITE | T1 | N0 | M0 |
| Group B | TCGA-A3-3378 | MALE | 60 | ccRCC | WHITE | T1 | N0 | M0 |
| Group B | TCGA-A3-3380 | MALE | 54 | ccRCC | NA | T1 | N0 | M0 |
| Group B | TCGA-B0-5104 | FEMALE | 90 | ccRCC | WHITE | T1 | N0 | M0 |
| Group B | TCGA-CZ-4859 | FEMALE | 59 | ccRCC | WHITE | T1 | N0 | M0 |
| Group B | TCGA-B0-5699 | MALE | 53 | ccRCC | WHITE | T1 | N0 | M0 |
| Group B | TCGA-B0-5705 | FEMALE | 65 | ccRCC | WHITE | T1 | N0 | M0 |
| Group B | TCGA-EU-5904 | FEMALE | 47 | ccRCC | WHITE | T1 | NX | M0 |
| Group B | TCGA-EU-5905 | FEMALE | 67 | ccRCC | WHITE | T1 | NX | M0 |
| Group B | TCGA-A3-3311 | MALE | 57 | ccRCC | NA | T1 | NX | M0 |
| Group B | TCGA-A3-3383 | MALE | 52 | ccRCC | WHITE | T1 | NX | M0 |
| Group B | TCGA-B0-5102 | FEMALE | 74 | ccRCC | WHITE | T1 | NX | M0 |
| Group B | TCGA-CZ-4866 | FEMALE | 79 | ccRCC | WHITE | T1 | NX | M0 |
| Group B | TCGA-B8-5545 | MALE | 42 | ccRCC | BLACK OR AFRICAN AMERICAN | T1a | N0 | M0 |
| Group B | TCGA-CJ-6030 | MALE | 65 | ccRCC | WHITE | T1a | N0 | M0 |
| Group B | TCGA-A3-3362 | FEMALE | 60 | ccRCC | WHITE | T1a | N0 | M0 |
| Group B | TCGA-B8-4148 | FEMALE | 63 | ccRCC | WHITE | T1a | N0 | M0 |
| Group B | TCGA-B8-4154 | FEMALE | 73 | ccRCC | WHITE | T1a | N0 | M0 |
| Group B | TCGA-A3-3376 | MALE | 51 | ccRCC | BLACK OR AFRICAN AMERICAN | T1a | N0 | M0 |
| Group B | TCGA-A3-3385 | FEMALE | 46 | ccRCC | WHITE | T1a | N0 | M0 |
| Group B | TCGA-B0-4823 | MALE | 88 | ccRCC | WHITE | T1a | N0 | M0 |
| Group B | TCGA-B0-4945 | FEMALE | 75 | ccRCC | WHITE | T1a | N0 | M0 |
| Group B | TCGA-B0-5106 | MALE | 64 | ccRCC | WHITE | T1a | N0 | M0 |
| Group B | TCGA-B0-5110 | FEMALE | 71 | ccRCC | WHITE | T1a | N0 | M0 |
| Group B | TCGA-B0-5120 | FEMALE | 72 | ccRCC | WHITE | T1a | N0 | M0 |
| Group B | TCGA-B8-5159 | FEMALE | 61 | ccRCC | WHITE | T1a | N0 | M0 |
| Group B | TCGA-B8-5165 | MALE | 43 | ccRCC | WHITE | T1a | N0 | M0 |
| Group B | TCGA-B0-5077 | MALE | 77 | ccRCC | WHITE | T1a | N0 | M0 |
| Group B | TCGA-BP-4964 | FEMALE | 54 | ccRCC | WHITE | T1a | N0 | M0 |
| Group B | TCGA-BP-4986 | MALE | 75 | ccRCC | WHITE | T1a | N0 | M0 |
| Group B | TCGA-BP-4988 | MALE | 72 | ccRCC | WHITE | T1a | N0 | M0 |
| Group B | TCGA-BP-5006 | MALE | 61 | ccRCC | WHITE | T1a | N0 | M0 |
| Group B | TCGA-BP-5182 | MALE | 56 | ccRCC | WHITE | T1a | N0 | M0 |
| Group B | TCGA-BP-5186 | FEMALE | 50 | ccRCC | WHITE | T1a | N0 | M0 |
| Group B | TCGA-A3-3358 | FEMALE | 57 | ccRCC | WHITE | T1a | N0 | M0 |
| Group B | TCGA-A3-3387 | MALE | 49 | ccRCC | WHITE | T1a | N0 | M0 |
| Group B | TCGA-B0-5691 | FEMALE | 66 | ccRCC | WHITE | T1a | N0 | M0 |
| Group B | TCGA-B0-5697 | MALE | 50 | ccRCC | WHITE | T1a | N0 | M0 |
| Group B | TCGA-B0-5707 | FEMALE | 39 | ccRCC | WHITE | T1a | N0 | M0 |
| Group B | TCGA-B2-5641 | MALE | 79 | ccRCC | WHITE | T1a | N0 | M0 |
| Group B | TCGA-CJ-6027 | MALE | 77 | ccRCC | WHITE | T1a | NX | M0 |
| Group B | TCGA-CW-6093 | MALE | 73 | ccRCC | WHITE | T1a | NX | M0 |
| Group B | TCGA-CZ-5982 | FEMALE | 59 | ccRCC | WHITE | T1a | NX | M0 |
| Group B | TCGA-A3-3322 | MALE | 51 | ccRCC | WHITE | T1a | NX | M0 |
| Group B | TCGA-A3-3326 | MALE | 47 | ccRCC | WHITE | T1a | NX | M0 |
| Group B | TCGA-A3-3365 | MALE | 46 | ccRCC | WHITE | T1a | NX | M0 |
| Group B | TCGA-AS-3778 | MALE | 35 | ccRCC | WHITE | T1a | NX | M0 |
| Group B | TCGA-B2-4099 | MALE | 83 | ccRCC | WHITE | T1a | NX | M0 |
| Group B | TCGA-BP-4782 | FEMALE | 55 | ccRCC | WHITE | T1a | NX | M0 |
| Group B | TCGA-BP-4801 | MALE | 57 | ccRCC | WHITE | T1a | NX | M0 |
| Group B | TCGA-BP-4993 | MALE | 58 | ccRCC | WHITE | T1a | NX | M0 |
| Group B | TCGA-BP-5168 | MALE | 75 | ccRCC | WHITE | T1a | NX | M0 |
| Group B | TCGA-BP-4961 | MALE | 47 | ccRCC | WHITE | T1a | NX | M0 |
| Group B | TCGA-BP-4976 | MALE | 77 | ccRCC | WHITE | T1a | NX | M0 |
| Group B | TCGA-BP-4991 | MALE | 54 | ccRCC | WHITE | T1a | NX | M0 |
| Group B | TCGA-BP-4998 | MALE | 49 | ccRCC | ASIAN | T1a | NX | M0 |
| Group B | TCGA-BP-4999 | MALE | 56 | ccRCC | WHITE | T1a | NX | M0 |
| Group B | TCGA-BP-5004 | MALE | 53 | ccRCC | WHITE | T1a | NX | M0 |
| Group B | TCGA-BP-5008 | MALE | 46 | ccRCC | WHITE | T1a | NX | M0 |
| Group B | TCGA-BP-5170 | MALE | 55 | ccRCC | WHITE | T1a | NX | M0 |
| Group B | TCGA-BP-5173 | MALE | 75 | ccRCC | WHITE | T1a | NX | M0 |
| Group B | TCGA-BP-5174 | FEMALE | 45 | ccRCC | WHITE | T1a | NX | M0 |
| Group B | TCGA-BP-5175 | MALE | 60 | ccRCC | WHITE | T1a | NX | M0 |
| Group B | TCGA-BP-5176 | FEMALE | 78 | ccRCC | WHITE | T1a | NX | M0 |
| Group B | TCGA-BP-5177 | FEMALE | 46 | ccRCC | WHITE | T1a | NX | M0 |
| Group B | TCGA-BP-5180 | MALE | 53 | ccRCC | WHITE | T1a | NX | M0 |
| Group B | TCGA-BP-5184 | MALE | 54 | ccRCC | WHITE | T1a | NX | M0 |
| Group B | TCGA-BP-5185 | MALE | 56 | ccRCC | WHITE | T1a | NX | M0 |
| **Sorted Group** | **Patient barcode** | **Sex** | **Age at diagnosis** | **Histological type** | **Race** | **Stage** | **Pathologic_N** | **Pathologic_M** |
| Group B | TCGA-BP-5187 | MALE | 54 | ccRCC | WHITE | T1a | NX | M0 |
| Group B | TCGA-BP-5190 | MALE | 61 | ccRCC | WHITE | T1a | NX | M0 |
| Group B | TCGA-BP-5192 | MALE | 59 | ccRCC | WHITE | T1a | NX | M0 |
| Group B | TCGA-BP-5194 | MALE | 39 | ccRCC | WHITE | T1a | NX | M0 |
| Group B | TCGA-BP-5195 | MALE | 75 | ccRCC | WHITE | T1a | NX | M0 |
| Group B | TCGA-BP-5196 | MALE | 53 | ccRCC | WHITE | T1a | NX | M0 |
| Group B | TCGA-CJ-4905 | FEMALE | 62 | ccRCC | WHITE | T1a | NX | M0 |
| Group B | TCGA-CJ-4908 | MALE | 38 | ccRCC | WHITE | T1a | NX | M0 |
| Group B | TCGA-CZ-4853 | MALE | 82 | ccRCC | WHITE | T1a | NX | M0 |
| Group B | TCGA-B2-5635 | MALE | 74 | ccRCC | WHITE | T1a | NX | M0 |
| Group B | TCGA-CJ-5671 | MALE | 51 | ccRCC | WHITE | T1a | NX | M0 |
| Group B | TCGA-CJ-5672 | MALE | 84 | ccRCC | WHITE | T1a | NX | M0 |
| Group B | TCGA-CW-5583 | FEMALE | 51 | ccRCC | WHITE | T1a | NX | M0 |
| Group B | TCGA-CW-5588 | FEMALE | 78 | ccRCC | WHITE | T1a | NX | M0 |
| Group B | TCGA-CW-5589 | MALE | 52 | ccRCC | WHITE | T1a | NX | M0 |
| Group B | TCGA-B0-5698 | MALE | 77 | ccRCC | BLACK OR AFRICAN AMERICAN | T1b | N0 | M0 |
| Group B | TCGA-B0-5710 | MALE | 57 | ccRCC | WHITE | T1b | N0 | M0 |
| Group B | TCGA-CZ-5984 | MALE | 51 | ccRCC | WHITE | T1b | N0 | M0 |
| Group B | TCGA-CZ-5988 | MALE | 38 | ccRCC | WHITE | T1b | N0 | M0 |
| Group B | TCGA-A3-3349 | FEMALE | 34 | ccRCC | WHITE | T1b | N0 | M0 |
| Group B | TCGA-BP-4162 | FEMALE | 65 | ccRCC | WHITE | T1b | N0 | M0 |
| Group B | TCGA-A3-3367 | MALE | 72 | ccRCC | WHITE | T1b | N0 | M0 |
| Group B | TCGA-A3-3370 | FEMALE | 48 | ccRCC | WHITE | T1b | N0 | M0 |
| Group B | TCGA-A3-3373 | FEMALE | 54 | ccRCC | WHITE | T1b | N0 | M0 |
| Group B | TCGA-B0-5119 | FEMALE | 61 | ccRCC | WHITE | T1b | N0 | M0 |
| Group B | TCGA-B0-5121 | MALE | 56 | ccRCC | WHITE | T1b | N0 | M0 |
| Group B | TCGA-B0-5088 | MALE | 53 | ccRCC | WHITE | T1b | N0 | M0 |
| Group B | TCGA-BP-4968 | MALE | 40 | ccRCC | WHITE | T1b | N0 | M0 |
| Group B | TCGA-BP-4995 | MALE | 68 | ccRCC | WHITE | T1b | N0 | M0 |
| Group B | TCGA-BP-5169 | MALE | 70 | ccRCC | WHITE | T1b | N0 | M0 |
| Group B | TCGA-CZ-4856 | FEMALE | 62 | ccRCC | WHITE | T1b | N0 | M0 |
| Group B | TCGA-B0-5399 | MALE | 46 | ccRCC | WHITE | T1b | N0 | M0 |
| Group B | TCGA-B8-4621 | MALE | 63 | ccRCC | BLACK OR AFRICAN AMERICAN | T1b | N0 | M0 |
| Group B | TCGA-B0-5695 | FEMALE | 61 | ccRCC | WHITE | T1b | N0 | M0 |
| Group B | TCGA-B0-5702 | MALE | 71 | ccRCC | WHITE | T1b | N0 | M0 |
| Group B | TCGA-B0-5703 | MALE | 73 | ccRCC | WHITE | T1b | N0 | M0 |
| Group B | TCGA-B2-5633 | MALE | 56 | ccRCC | WHITE | T1b | N0 | M0 |
| Group B | TCGA-B8-5546 | FEMALE | 38 | ccRCC | BLACK OR AFRICAN AMERICAN | T1b | N0 | M0 |
| Group B | TCGA-B8-5549 | MALE | 53 | ccRCC | WHITE | T1b | N0 | M0 |
| Group B | TCGA-B8-5551 | FEMALE | 65 | ccRCC | BLACK OR AFRICAN AMERICAN | T1b | N0 | M0 |
| Group B | TCGA-B0-5812 | MALE | 53 | ccRCC | WHITE | T1b | NX | M0 |
| Group B | TCGA-B8-4146 | FEMALE | 41 | ccRCC | WHITE | T1b | NX | M0 |
| Group B | TCGA-B8-5552 | FEMALE | 41 | ccRCC | WHITE | T1b | NX | M0 |
| Group B | TCGA-CJ-5686 | FEMALE | 59 | ccRCC | WHITE | T1b | NX | M0 |
| Group B | TCGA-CJ-6031 | MALE | 54 | ccRCC | WHITE | T1b | NX | M0 |
| Group B | TCGA-CW-6090 | MALE | 68 | ccRCC | WHITE | T1b | NX | M0 |
| Group B | TCGA-EU-5906 | MALE | 55 | ccRCC | WHITE | T1b | NX | M0 |
| Group B | TCGA-A3-3319 | MALE | 70 | ccRCC | WHITE | T1b | NX | M0 |
| Group B | TCGA-A3-3320 | FEMALE | 52 | ccRCC | WHITE | T1b | NX | M0 |
| Group B | TCGA-A3-3323 | MALE | 53 | ccRCC | WHITE | T1b | NX | M0 |
| Group B | TCGA-A3-3324 | MALE | 51 | ccRCC | WHITE | T1b | NX | M0 |
| Group B | TCGA-A3-3346 | MALE | 68 | ccRCC | WHITE | T1b | NX | M0 |
| Group B | TCGA-A3-3382 | MALE | 69 | ccRCC | WHITE | T1b | NX | M0 |
| Group B | TCGA-AK-3444 | FEMALE | 80 | ccRCC | WHITE | T1b | NX | M0 |
| Group B | TCGA-B2-3924 | MALE | 73 | ccRCC | WHITE | T1b | NX | M0 |
| Group B | TCGA-B2-4098 | FEMALE | 72 | ccRCC | BLACK OR AFRICAN AMERICAN | T1b | NX | M0 |
| Group B | TCGA-B2-4102 | MALE | 61 | ccRCC | WHITE | T1b | NX | M0 |
| Group B | TCGA-BP-4161 | MALE | 74 | ccRCC | WHITE | T1b | NX | M0 |
| Group B | TCGA-CJ-4634 | FEMALE | 60 | ccRCC | WHITE | T1b | NX | M0 |
| Group B | TCGA-BP-4963 | MALE | 63 | ccRCC | WHITE | T1b | NX | M0 |
| Group B | TCGA-BP-4975 | MALE | 40 | ccRCC | WHITE | T1b | NX | M0 |
| Group B | TCGA-BP-4977 | MALE | 57 | ccRCC | WHITE | T1b | NX | M0 |
| Group B | TCGA-BP-4982 | MALE | 42 | ccRCC | WHITE | T1b | NX | M0 |
| Group B | TCGA-BP-4987 | FEMALE | 41 | ccRCC | ASIAN | T1b | NX | M0 |
| Group B | TCGA-BP-4992 | MALE | 66 | ccRCC | WHITE | T1b | NX | M0 |
| Group B | TCGA-BP-5000 | MALE | 40 | ccRCC | WHITE | T1b | NX | M0 |
| Group B | TCGA-BP-5001 | FEMALE | 43 | ccRCC | WHITE | T1b | NX | M0 |
| Group B | TCGA-BP-5009 | MALE | 52 | ccRCC | WHITE | T1b | NX | M0 |
| Group B | TCGA-CJ-4899 | MALE | 42 | ccRCC | ASIAN | T1b | NX | M0 |
| Group B | TCGA-BP-5189 | MALE | 60 | ccRCC | WHITE | T1b | NX | M0 |
| Group B | TCGA-CJ-4903 | MALE | 50 | ccRCC | WHITE | T1b | NX | M0 |
| **Sorted Group** | **Patient barcode** | **Sex** | **Age at diagnosis** | **Histological type** | **Race** | **Stage** | **Pathologic_N** | **Pathologic_M** |
| Group B | TCGA-CJ-4920 | FEMALE | 64 | ccRCC | WHITE | T1b | NX | M0 |
| Group B | TCGA-AK-3458 | MALE | 48 | ccRCC | WHITE | T1b | NX | M0 |
| Group B | TCGA-B0-5693 | FEMALE | 47 | ccRCC | WHITE | T1b | NX | M0 |
| Group B | TCGA-CJ-5683 | MALE | 78 | ccRCC | WHITE | T1b | NX | M0 |
| Group B | TCGA-CW-5581 | MALE | 44 | ccRCC | WHITE | T1b | NX | M0 |
| Group C | TCGA-B0-5713 | FEMALE | 75 | ccRCC | WHITE | T3b | N0 | M0 |
| Group C | TCGA-A3-3308 | FEMALE | 77 | ccRCC | WHITE | T3b | N0 | M0 |
| Group C | TCGA-B0-5097 | FEMALE | 59 | ccRCC | WHITE | T3b | N0 | M0 |
| Group C | TCGA-B0-5116 | MALE | 52 | ccRCC | WHITE | T3b | N0 | M0 |
| Group C | TCGA-B0-5081 | FEMALE | 79 | ccRCC | WHITE | T3b | N0 | M0 |
| Group C | TCGA-BP-5198 | MALE | 72 | ccRCC | WHITE | T3b | N0 | M0 |
| Group C | TCGA-B0-5400 | FEMALE | 59 | ccRCC | WHITE | T3b | N0 | M0 |
| Group C | TCGA-CZ-4863 | FEMALE | 51 | ccRCC | WHITE | T3b | N0 | M0 |
| Group C | TCGA-B0-5692 | FEMALE | 66 | ccRCC | WHITE | T3b | N0 | M0 |
| Group C | TCGA-B0-5701 | MALE | 65 | ccRCC | WHITE | T3b | N0 | M0 |
| Group C | TCGA-B0-5711 | MALE | 50 | ccRCC | WHITE | T3b | NX | M0 |
| Group C | TCGA-CJ-4901 | MALE | 47 | ccRCC | WHITE | T3b | NX | M0 |
| Group C | TCGA-CJ-4907 | MALE | 58 | ccRCC | WHITE | T3b | NX | M0 |
| Group C | TCGA-CZ-5459 | MALE | 63 | ccRCC | WHITE | T3b | NX | M0 |
| Group C | TCGA-CZ-5465 | FEMALE | 76 | ccRCC | NA | T3b | NX | M0 |
| Group C | TCGA-CJ-5676 | MALE | 47 | ccRCC | WHITE | T3b | NX | M0 |
| Group D | TCGA-B4-5377 | FEMALE | 68 | ccRCC | WHITE | T3 | N0 | M1 |
| Group D | TCGA-B0-5096 | FEMALE | 72 | ccRCC | WHITE | T3a | N1 | M0 |
| Group D | TCGA-B8-5158 | MALE | 56 | ccRCC | WHITE | T3a | N1 | M0 |
| Group D | TCGA-B0-4810 | MALE | 47 | ccRCC | WHITE | T3a | N1 | M0 |
| Group D | TCGA-CJ-6033 | FEMALE | 54 | ccRCC | WHITE | T3a | N0 | M1 |
| Group D | TCGA-B8-4143 | FEMALE | 66 | ccRCC | WHITE | T3a | N0 | M1 |
| Group D | TCGA-CJ-4644 | FEMALE | 48 | ccRCC | WHITE | T3a | N0 | M1 |
| Group D | TCGA-B8-4622 | MALE | 57 | ccRCC | WHITE | T3a | N0 | M1 |
| Group D | TCGA-BP-4974 | MALE | 58 | ccRCC | WHITE | T3a | N0 | M1 |
| Group D | TCGA-CJ-4918 | MALE | 64 | ccRCC | WHITE | T3a | N0 | M1 |
| Group D | TCGA-B0-5080 | MALE | 63 | ccRCC | WHITE | T3a | N0 | M1 |
| Group D | TCGA-CW-5591 | MALE | 56 | ccRCC | WHITE | T3a | N0 | M1 |
| Group D | TCGA-CW-6087 | MALE | 61 | ccRCC | WHITE | T3a | N1 | M1 |
| Group D | TCGA-CJ-4638 | FEMALE | 46 | ccRCC | WHITE | T3a | N1 | M1 |
| Group D | TCGA-CJ-4904 | FEMALE | 60 | ccRCC | WHITE | T3a | N1 | M1 |
| Group D | TCGA-CJ-6028 | MALE | 58 | ccRCC | WHITE | T3a | NX | M1 |
| Group D | TCGA-CW-5580 | FEMALE | 73 | ccRCC | WHITE | T3a | NX | M1 |
| Group D | TCGA-CJ-4641 | FEMALE | 55 | ccRCC | WHITE | T3a | NX | M1 |
| Group D | TCGA-BP-5178 | MALE | 71 | ccRCC | WHITE | T3a | NX | M1 |
| Group D | TCGA-CJ-4923 | FEMALE | 63 | ccRCC | WHITE | T3a | NX | M1 |
| Group D | TCGA-B0-4712 | MALE | 76 | ccRCC | WHITE | T3a | NX | M1 |
| Group D | TCGA-CJ-5677 | FEMALE | 54 | ccRCC | WHITE | T3a | NX | M1 |
| Group D | TCGA-CJ-5680 | FEMALE | 65 | ccRCC | WHITE | T3a | NX | M1 |
| Group D | TCGA-CJ-5681 | FEMALE | 44 | ccRCC | WHITE | T3a | NX | M1 |
| Group D | TCGA-CJ-5682 | MALE | 60 | ccRCC | WHITE | T3a | NX | M1 |
| Group D | TCGA-AK-3430 | MALE | 61 | ccRCC | WHITE | T3b | N1 | M0 |
| Group D | TCGA-B0-5109 | MALE | 69 | ccRCC | WHITE | T3b | N1 | M0 |
| Group D | TCGA-CW-5584 | MALE | 74 | ccRCC | WHITE | T3b | N1 | M0 |
| Group D | TCGA-B0-5094 | MALE | 62 | ccRCC | WHITE | T3b | N0 | M1 |
| Group D | TCGA-BP-5201 | MALE | 63 | ccRCC | WHITE | T3b | N0 | M1 |
| Group D | TCGA-CW-5585 | MALE | 51 | ccRCC | WHITE | T3b | N0 | M1 |
| Group D | TCGA-CZ-5987 | MALE | 60 | ccRCC | WHITE | T3b | NX | M1 |
| Group D | TCGA-CZ-5460 | MALE | 55 | ccRCC | WHITE | T3b | NX | M1 |
| Group D | TCGA-CZ-5464 | MALE | 69 | ccRCC | WHITE | T3b | NX | M1 |
| Group D | TCGA-CZ-5468 | MALE | 84 | ccRCC | WHITE | T3b | NX | M1 |
| Group D | TCGA-B0-4700 | MALE | 60 | ccRCC | WHITE | T4 | NX | M1 |
